# Laser induced temperature-jump time resolved IR spectroscopy of zeolites†

**DOI:** 10.1039/d3sc06128k

**Published:** 2024-02-07

**Authors:** Alexander P. Hawkins, Amy E. Edmeades, Christopher D. M. Hutchison, Michael Towrie, Russell F. Howe, Gregory M. Greetham, Paul M. Donaldson

**Affiliations:** a Central Laser Facility, Research Complex at Harwell, STFC Rutherford Appleton Laboratory Didcot Oxon OX11 0QX UK paul.donaldson@stfc.ac.uk; b Department of Chemistry, University of Aberdeen Aberdeen AB24 3UE UK

## Abstract

Combining pulsed laser heating and time-resolved infrared (TR-IR) absorption spectroscopy provides a means of initiating and studying thermally activated chemical reactions and diffusion processes in heterogeneous catalysts on timescales from nanoseconds to seconds. To this end, we investigated single pulse and burst laser heating in zeolite catalysts under realistic conditions using TR-IR spectroscopy. 1 ns, 70 μJ, 2.8 μm laser pulses from a Nd:YAG-pumped optical parametric oscillator were observed to induce temperature-jumps (T-jumps) in zeolite pellets in nanoseconds, with the sample cooling over 1–3 ms. By adopting a tightly focused beam geometry, T-jumps as large as 145 °C from the starting temperature were achieved, demonstrated through comparison of the TR-IR spectra with temperature dependent IR absorption spectra and three dimensional heat transfer modelling using realistic experimental parameters. The simulations provide a detailed understanding of the temperature distribution within the sample and its evolution over the cooling period, which we observe to be bi-exponential. These results provide foundations for determining the magnitude of a T-jump in a catalyst/adsorbate system from its absorption spectrum and physical properties, and for applying T-jump TR-IR spectroscopy to the study of reactive chemistry in heterogeneous catalysts.

## Abstract 2

Laser heating of zeolites in combination with time-resolved IR spectroscopy is introduced as a means of studying chemical processes in heterogeneous catalysts from nanoseconds to seconds.

## Graphical abstract

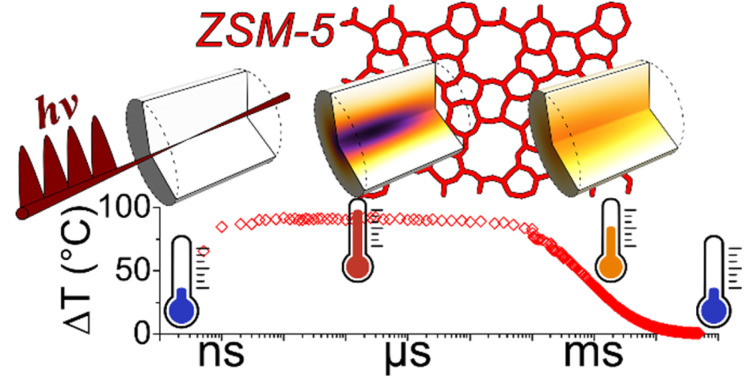

## Introduction

Zeolites are aluminosilicate materials whose nanoporous structure leads to wide application as shape-selective catalysts, catalytic supports and separation materials across multiple chemical industry sectors.^1,2^ Catalytic activity occurs at either incorporated metal centres^3^ or at framework Brønsted acid sites in the case of protonated zeolites.^1^ These applications depend on the complex geometric and chemical properties of the zeolite pore network, dictating how molecules adsorb into and diffuse through the material. Studying the chemistry and interactions of molecules in zeolites on timescales relevant to their diffusion and reaction is critical for developing an improved understanding of the many mechanisms of catalysis in these important materials.

The goal of the work in this paper is the development of methods of IR laser spectroscopy that can initiate and probe chemical change in zeolites across timescales of nanoseconds to seconds. Pulsed-laser infrared (IR) absorption spectroscopy allows vibrational spectra to be recorded with a time resolution as small as femtoseconds,^4,5^ allowing the study of dynamical processes such as bond breaking, structural rearrangements and molecular diffusion.^6–9^ Dynamical processes are initiated by pulsed-laser excitation and monitored with synchronised, time-resolved spectroscopic probing of the resulting molecular changes. It is common to initiate chemistry by excitation of electronic absorption bands in the UV/visible part of the spectrum, however using a laser to simply heat a sample (temperature-jump, or T-jump spectroscopy) enables thermally activated processes to be studied over multiple decades in time from picoseconds to milliseconds.^10–14^ The time for a sample to fall back to its initial temperature is dependent on its thermal properties. Starting with ps–ns heating pulses, elevated temperatures in the millisecond range are possible for solution phase samples, allowing non-equilibrium structural dynamics to be observed,^14–16^ with further timescales accessible through more sophisticated laser heater sources.^15^

While IR laser T-jump methods have seen application to solution-phase chemistry^10–19^ particularly in the field of biomolecule dynamics,^10–13,16,18,19^ time resolved IR T-jump investigations of heterogeneous catalysts are absent in the literature. The use of CW lasers to simply heat zeolites has been explored,^20–22^ but the ability to synchronously collect time-resolved spectra on the evolution of the sample was not implemented. Picosecond pulsed-IR investigations of zeolites have demonstrated how the relaxation of excited O–H and O–D vibrations induces heating of the zeolite framework, and quantified the resulting temperature transients, but considered the heating as an unwanted background to the investigation of vibrational relaxation on picosecond timescales.^23,24^ The ability to heat solid materials and monitor their evolution from nanoseconds to seconds with time-resolved IR remains unexplored. In this paper we describe experiments to do exactly this with an approach of pulsed-laser T-jumping and time-resolved IR probing for investigating laser heating in a hydrated commercial H-ZSM-5 zeolite powder. Acid zeolites possess strongly absorbing O–H vibrations at around 3600 cm^−1^. These are ideal modes to optically pump and induce the required laser heating. To this end, a Nd:YAG-pumped Optical Parametric Oscillator (OPO) operating in the mid-IR was used. Time resolved multiple probe spectroscopy (TRMPS) with a broadband IR probe^14^ was able to access dynamical timescales from nanoseconds to seconds in a single measurement, representing a new approach to investigate fast temperature-related phenomena in zeolites. We demonstrate here time-resolved IR experiments performed in transmission with compressed (pelleted) solid zeolite samples under conditions of controlled gas flow and elevated sample temperature. Classical heat transfer simulations^14,15^ of the pumped sample are used to provide insight into the spatial distribution and temporal evolution of heat. We experimentally determine the time-dependent temperature of the laser heated zeolite sample relative to its starting conditions by using the established approach of identifying temperature-dependant modes in the IR spectrum for use as spectroscopic ‘thermometers’. For zeolites, by partially deuterating the samples and probing spectral changes in the 2500–3000 cm^−1^ spectral range, we find that the narrow, temperature-sensitive stretch band of deuterated, intrinsic silanol groups makes an excellent ‘thermometer’. Optical scattering from the bright T-jump laser pulse can easily saturate the IR detector used to monitor the probe transmission. Partial deuteration allows *ν*(OH) stretch mode pumping and probing in the separate spectral region of mainly *ν*(OD) stretch modes.

Compared with earlier liquid-phase T-jump studies,^14,15^ we find that a significant increase in T-jump magnitude can be gained by tightly focussing the pump beam to radial dimensions of ∼40 microns in size. An even tighter focussed probe beam then passes through the centre of the excited sample volume. Such tightly focussed ns excitation pulses can cause cavitation and shock waves in liquid sample cells. A significant observation in this work is that zeolites, in the form of free-standing, compressed pellets of microcrystalline particles are robust under such excitation– we observed no evidence of cavitation, shock waves or damage. The lower thermal conductivity and specific heat capacity of zeolites compared to liquid water also means that larger T-jumps for a given energy input are achieved. Comparison of time resolved silanol *ν*(OD) stretch shifts with steady-state Fourier transform IR (FT-IR) spectra confirm that single laser pulse T-jumps with magnitudes of up to +145 °C above starting temperature are achieved with lifetimes into the millisecond time range. Allowing the Nd:YAG OPO pump laser to release repeated bursts of heating pulses spaced 1 ms apart achieves even higher levels of heating. These results establish an experimental and conceptual framework for the measurement and analysis of T-jumps which we expect to be applicable to a wide variety of zeolite-adsorbate systems.

## Experimental

### Samples

ZSM-5 was sourced from Zeolyst (CBV5524G) in the NH_4_-ZSM-5 powder form and converted to H-ZSM-5 by calcination in static air at 550 °C for 24 hours. X-ray fluorescence characterisation of the calcined powder confirmed a nominal framework Si : Al ratio of 25 : 1.

All spectroscopic measurements were performed in transmission geometry on self-supporting zeolite pellets of 70–150 μm thickness prepared from 7–15 mg of powdered sample held at 1 ton for 1 minute in a pellet press (Specac). Pellets were held in a heated spectroscopy cell with gas-flow capability (Linkam FTIR600) and sample temperature controlled by the sample being in direct contact with a cell heater block held between (but not in contact with) two CaF_2_ windows. A centrally located 4 mm hole in the heater block allowed passage of light through the pellet. For a given heater block temperature set-point, the free-standing portion of the zeolite had a static thermal gradient, with the sample coolest in the middle (farthest from the heater block). This gradient was measured by thermal imaging of the sample.^25^

Measurements were carried out at 1 atmosphere under flowing N_2_ (liquid N_2_ boil-off) with sample hydration and deuteration achieved by sparging controlled flows of N_2_ through a Drechsel flask containing a 20% solution of D_2_O in H_2_O (referred to as HOD/H_2_O below for brevity). A schematic of the gas flow apparatus is shown in the ESI Section 1.† FT-IR spectra were collected using a Bruker Tensor spectrometer operated at 4 cm^−1^ spectral resolution.

### Time-resolved spectroscopy

**Fig. 1:**
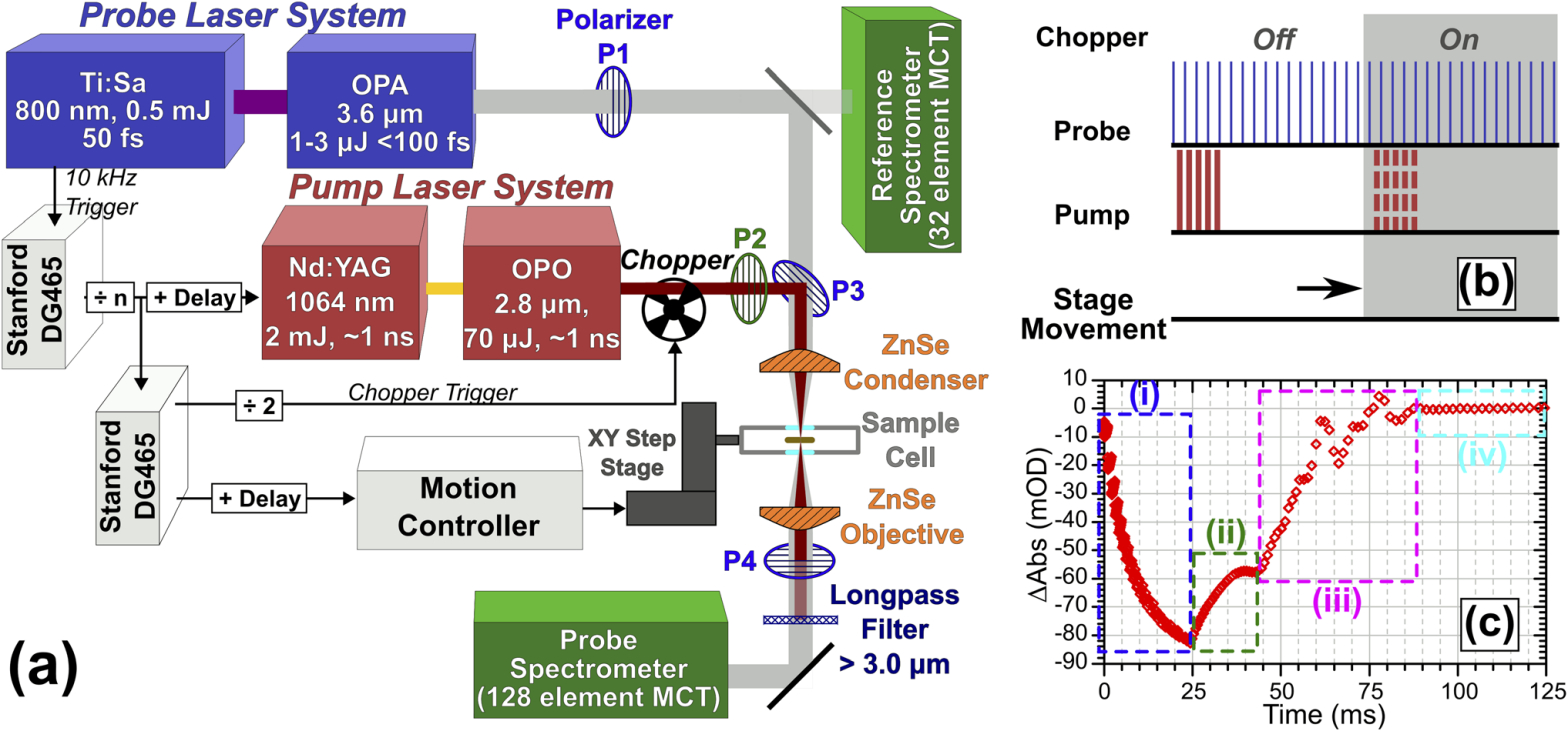
(a) Experimental layout showing optical and sample environment components for time-resolved IR spectroscopy T-jump measurements. (b) Pulse scheme and synchronised sample movement for TRMPS technique. (c) Spectral intensity at 2750 cm^−1^*vs.* time for HOD/H_2_O in ZSM-5 excited with 25 × 70 μJ pump pulses showing effects of (i) sample excitation, (ii) decay, (iii) noise from stage movement and (iv) return to unpumped state post-movement.

T-Jump spectroscopy was carried out at the Rutherford-Appleton Laboratory Ultra facility using a variation on the TRMPS technique.^14,26^ The experiment is shown in Fig. 1. To adequately cover the *ν*(CH) and *ν*(OD) modes of the vibrational spectrum, probe pulses centred at *ca.* 2700 cm^−1^ with a useable bandwidth >500 cm^−1^ and an energy of 1–2 μJ per pulse were generated at 10 kHz by difference frequency mixing the output of an Optical Parametric Amplifier (OPA, Light Conversion TOPAS) pumped by 5 W of 800 nm light sourced from a 20 W, 50 fs Ti:Sapphire amplifier system (Coherent Legend Elite Duo). Pump pulses of ∼110 μJ energy and <1.5 ns duration centred at 3600 cm^−1^ were generated using 2 mJ of light from a Nd:YAG (BrightSolutions Wedge, 1 kHz, 4 mJ) pumping a home-built OPO based on periodically poled lithium niobate (PPLN, Covesion) heated to 75 °C to give an OPO output of 3600 cm^−1^ with a pulse duration <1.5 ns.^14^ In Fig. 1, the pump laser is synchronised to the probe laser and operated at repetition rates from 2 Hz to 1 kHz. High-voltage Q-switch noise from the pump laser coupling into the IR detection system is subtracted using a chopper operating at half the pump repetition rate, giving a ‘pump-off’ dataset for each entire TRMPS probe sequence.^14^ The longest TRMPS time delay measurable is then half the pump pulse period. The 10 kHz probing gives multiple probe pulses spaced by 100 μs. To achieve sub-100 μs time resolution on the first probe pulse of the sequence, the pump–probe pulse timing is controlled by triggering the Nd:YAG Q-switch at a fixed delay set by a computer controlled pulse delay generator (Stanford DG465), which could also trigger the Nd:YAG laser to make bursts of pulses, each burst treated like a single pump event. For the data in this paper, TRMPS spectra for a given electronic delay were averaged for 30–60 seconds. Further details regarding the triggering of the chopper, sample stage movement and pump bursts are described in ESI Section 2.†

The pump and probe beams were orthogonally polarised and combined collinearly on a wire grid polariser (P3 in Fig. 1), then focused onto the sample using a 12.7 mm focal length ZnSe asphere (Thorlabs). The profiles and divergence characteristics of the focussed beams were examined using a microbolometer camera at the focal plane (see ESI Section 3†). An accurate characterisation was critical for simulations. The average FWHM size of the pump and probe at the focal plane were 36 ± 1.4 and 21.5 ± 1 μm respectively. Pulse energies incident on the sample were 71 ± 5 μJ (pump) and <0.5 μJ (probe). After the sample, the beams were re-collimated with a second ZnSe asphere. Residual/scattered pump light was rejected with a long-pass filter. A grating spectrometer dispersed the probe light onto a 128-pixel HgCdTe detector (Infrared Associates) with ∼4 cm^−1^ resolution per-pixel. IR spectral intensities were digitised shot-by-shot (Infrared Development Systems FPAS) and correlated against simultaneous probe reference intensity measurements recorded using a small amount of pre-sample probe light sent to a separate spectrometer/detection system to correct for intensity fluctuations using a method similar to that of Feng *et al.*^27^

Spectra were recorded using one of two different measurement schemes. In the first, the sample was held stationary and the pump laser triggered at 20 Hz (chopped to 10 Hz), giving a data collection rate of 10 measurements per second and a 50 ms spectral collection window per measurement due to the need to chop the pump beam. To allow measurement of samples not fully returning to equilibrium conditions in <50 ms, the sample was mounted on a motorised *XY* translation stage (two Newport XMS series stages, see ESI Section 2†) which performed a triggered 200 μm step movement of the sample in <50 ms at the end of each measurement, ensuring that each T-jump occurred from an unpumped region of the sample. These step measurements were collected at a rate of 4 measurements per second, giving a 20–40 ms time window stationary interval post pumping while allowing for the movement time of the *XY* translation stage.

## Results and discussion

**Fig. 2:**
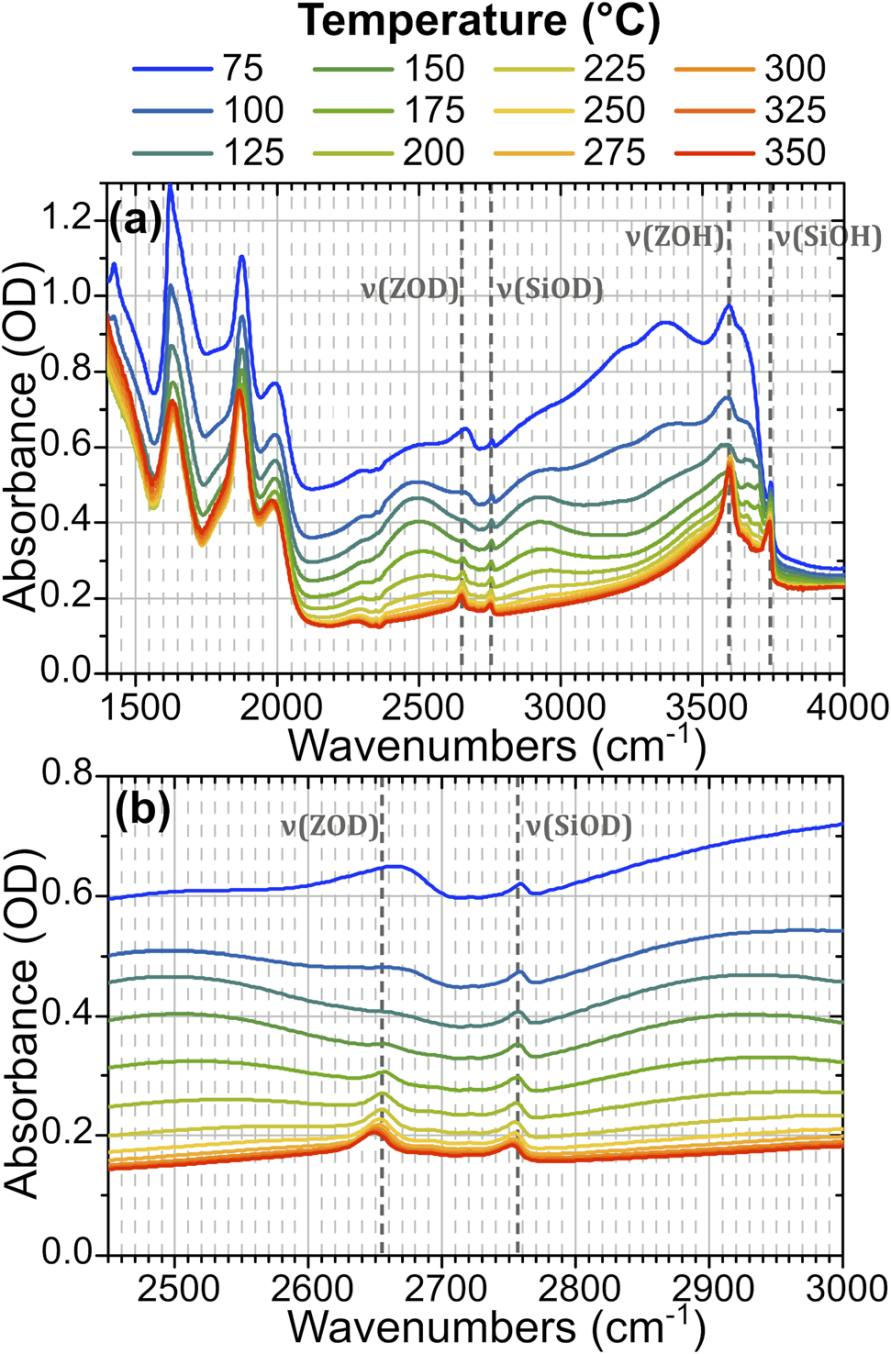
IR absorption (FTIR) spectra from 1400–4000 cm^−1^ of ZSM-5 held in flowing HOD/H_2_O vapour at indicated temperatures showing (a) all features and (b) the detection range used in the time-resolved spectroscopy. The location of *ν*(SiOH) silanol modes, free *ν*(ZOH) Brønsted acid modes and their deuterated equivalents are highlighted.

In order to understand the time-resolved T-jump data it is useful to first assign the features present in the steady-state FTIR spectra. The IR spectrum of hydrated ZSM-5 is well studied^28–31^ and Fig. 2 illustrates how the spectrum of the partially deuterium-exchanged zeolite changes across the temperature range investigated. The sharp feature at 2760 cm^−1^ corresponds to *ν*(SiOD) modes of silanol groups at the crystal exterior and in internal defects. It is present at all temperatures. The equivalent *ν*(SiOH) mode is visible at 3750 cm^−1^. Above 125 °C, the free *ν*(OH) Brønsted acid site band is visible at 3590 cm^−1^, and the equivalent *ν*(OD) band at 2655 cm^−1^. We refer to these bands as *ν*(ZOH) and *ν*(ZOD) respectively. Hydrogen bonding of ZOH with one or two adsorbed water molecules results in a substantial broadening, structuring and red shifting of the *ν*(ZOH) band.^29,32,33^ This creates a broad doublet peaking at *ca.* 2900 cm^−1^ and *ca.* 2500 cm^−1^. We refer to this band as *ν*(ZOH⋯(OH_2_)_*n*=1,2_) but note that only the 2900 cm^−1^ component of it is fully visible within the spectral range examined in the time-resolved experiments. The corresponding *ν*(ZOD⋯(OH_2_)_*n*=1,2_) band or bands are obscured by the zeolite lattice bands below 2100 cm^−1^. Below 125 °C the spectrum shifts in shape towards the form associated with water clusters with >2H_2_O molecules per Brønsted acid site, referred to as ZO^−^⋯H^+^(H_2_O)_*n*>2_.^28,31,34^ Note also that at higher water coverages (lower temperatures) *ν*(OH)/*ν*(OD) bands due to weakly hydrogen bonded (terminal) OH/OD groups in hydrogen bonded clusters occur at frequencies close to those of ZOH and ZOD. The weak peak at 2705 cm^−1^ at high temperatures is due to a small population of deuterium exchanged extra-framework aluminium hydroxide species within the pore network, a feature common in commercial zeolite materials.^35,36^

**Fig. 3:**
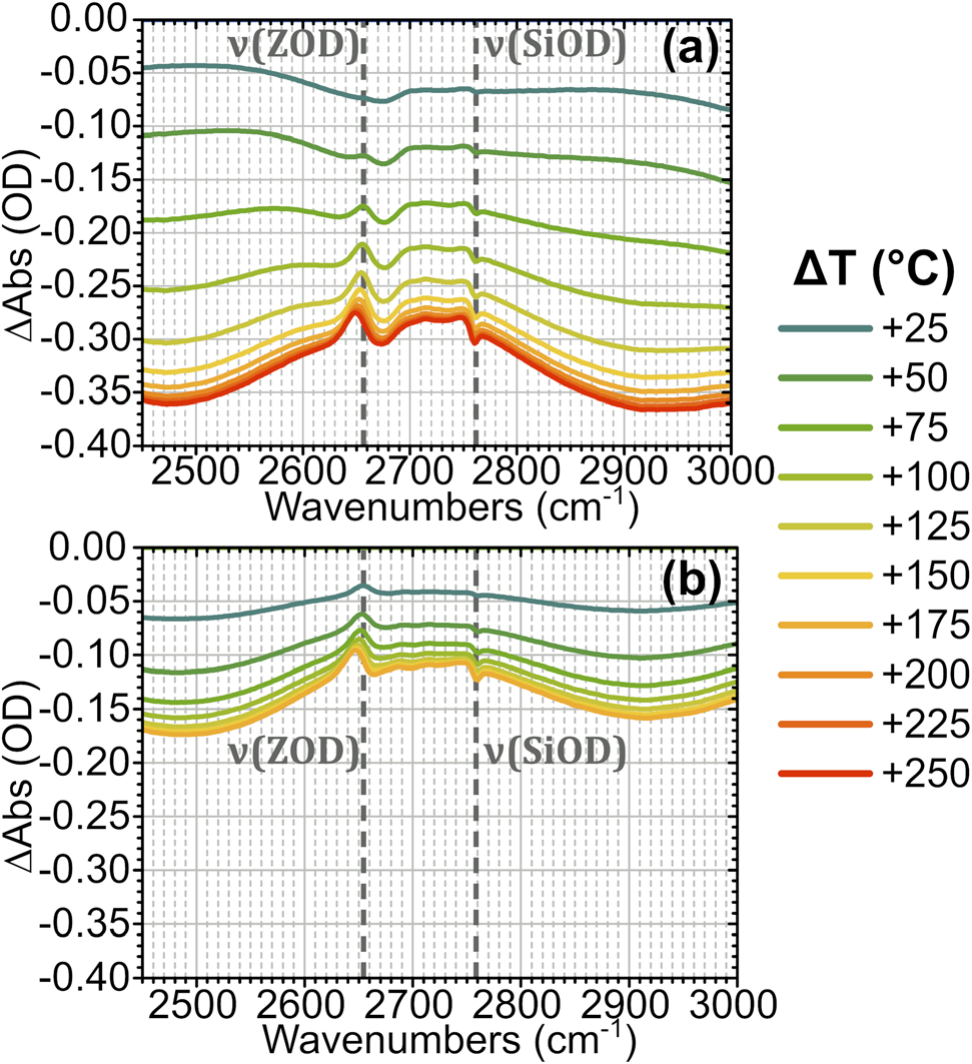
FTIR temperature ramp difference spectra of ZSM-5 in flowing HOD/H_2_O vapour on heating relative to 100 °C (a) and 175 °C (b) at the indicated Δ*T* values. The corresponding difference spectra relative to other temperatures are shown in the ESI, Section 4.† The location of *ν*(SiOD) silanol modes and *ν*(ZOD) Brønsted acid modes are highlighted.

Fig. 3 shows FTIR difference spectra centred on the time-resolved experiment region of hydrated ZSM-5 on heating from initial temperatures of 100 °C (a) and 175 °C (b). The spectra were collected as a continuous temperature ramp (2 °C per minute). We define the temperature difference Δ*T* = *T*_ramp_ − *T*_init_.

Removal of Brønsted site-associated hydrogen-bonded water results in negative intensity changes in the difference spectra in this region. At small Δ*T* values for the 100 °C *T*_Init_ sample it is mostly clustered water ZO^−^⋯H^+^(H_2_O)_*n*>2_ which is removed, giving a flatter difference spectrum (Fig. 3(a)). With increasing Δ*T*, at *T*_init_ = 100 °C, the removal of ZOH⋯(OH_2_)_*n*=1,2_ water molecules hydrogen-bonded to the protonated Brønsted sites results in broad negative features at ∼2900 and 2500 cm^−1^ in the difference spectra due to loss of the hydrogen bonds. Superimposed on these broad negative features is a positive feature at 2660 cm^−1^ due to Brønsted acid ZOD groups recovered by the water loss. Note that the shape of this feature is distorted by the temperature dependent frequency shift well known for zeolite hydroxyl groups.^37,38^ There is also a negative feature at the SiOD frequency (2760 cm^−1^) which increases with increasing temperature. This is due to the fact that the absorption coefficient for *ν*(SiOD) vibrations decreases markedly with increasing temperature, as discussed further below. For *T*_Init_ = 175 °C the sample contains no clustered water ZO^−^⋯H^+^(H_2_O)_*n*>2_ and the removal of ZOH⋯(OH_2_)_*n*=1,2_ water proceeds for all values of Δ*T* (Fig. 3(b)). Intensity changes are weaker in the *T*_Init_ = 175 °C spectrum for the same Δ*T* value due to the lower initial population of water.

**Fig. 4:**
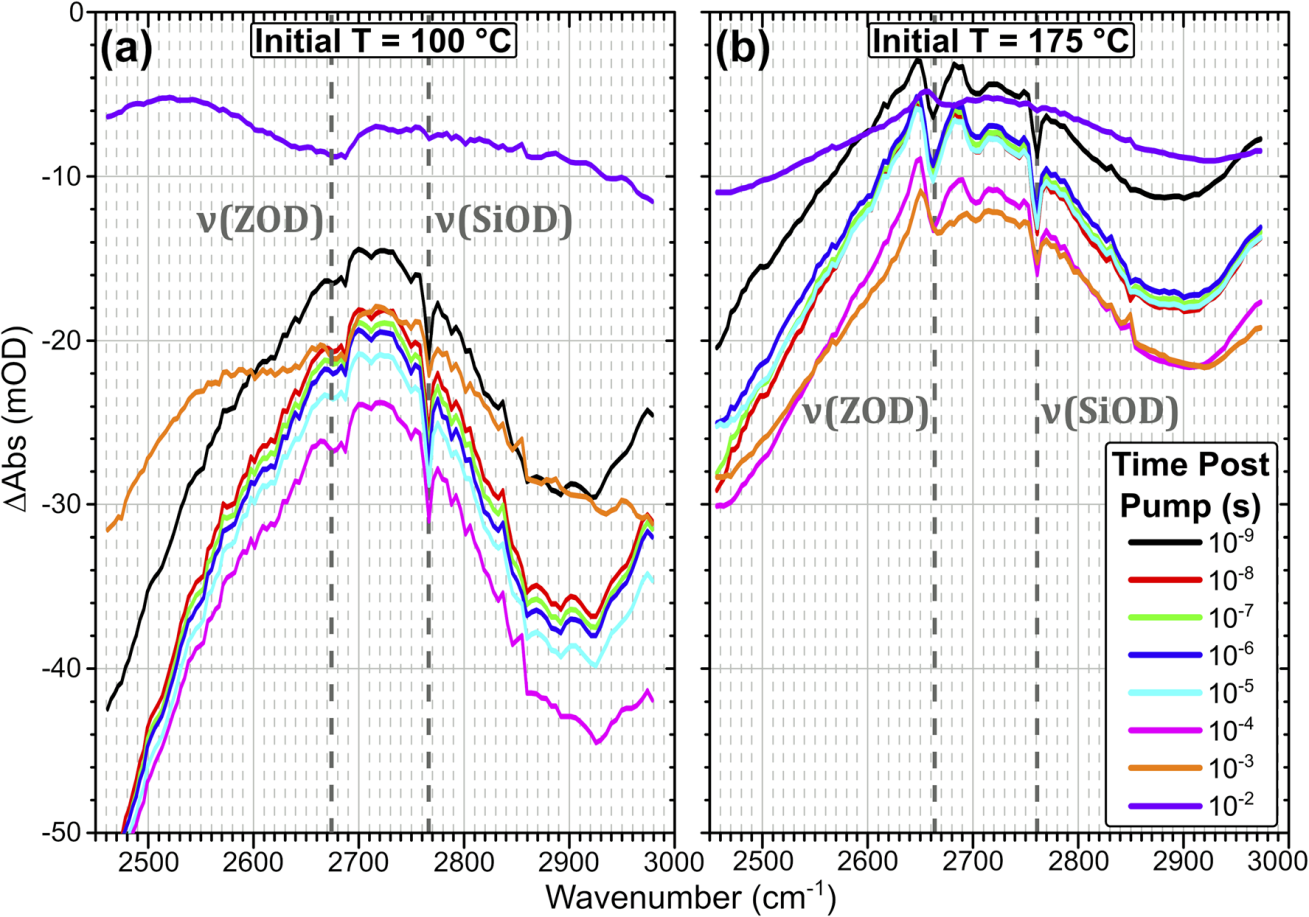
Time-resolved IR spectral evolution of ZSM-5 in flowing HOD/H_2_O vapour at an initial temperature of 100 °C (a) and 175 °C (b). Spectra are shown at logarithmic time intervals from 10^−9^–10^−2^ s following pumping with the 70 μJ laser pulse. The *ν*(SiOD) silanol modes and free *ν*(OD) Brønsted acid modes are highlighted.

Fig. 4 shows T-jump transient IR spectra across a time range of over seven orders of magnitude for a ZSM-5 pellet held in the presence of dilute HOD/H_2_O vapour and heated with a ns IR laser pulse at a 10 Hz repetition rate. Initial temperatures (*T*_Init_) of 100 and 175 °C are chosen for discussion as they are representative of the overall trends in sample response, and allow a direct comparison with the temperature ramp FTIR data in Fig. 3. Samples were measured at 8 initial temperatures from 80–300 °C. Transient IR spectra for these additional *T*_Init_ values are shown in ESI Section 5.† The plots are difference spectra (in units of optical density) relative to the unpumped state of the sample. Negative signals correspond to an increase in transmission (decrease in absorption), positive signals correspond to a decrease in transmission (increase in absorption).

The spectral changes observed in the T-jump transient spectra of Fig. 4 are consistent with the sample undergoing rapid heating due to absorption of the pump laser light, and qualitatively match the form of difference spectra taken by FT-IR for increasing temperature in Fig. 3. For the sample held initially at 100 °C (Fig. 4(a)) the loss of adsorbed water is seen as a negative baseline shift due to loss of water clusters, and the growth of two negative features at ∼2900 cm^−1^ and below 2500 cm^−1^ due to loss of hydrogen bonded ZOD groups, as discussed for the FT-IR temperature ramp. These negative changes are ∼5× smaller than those seen in the FTIR difference spectra, presumably because in the T-jump experiments the sample begins cooling before desorption reaches equilibrium. The subsequent decline of these negative features indicates that water readsorbs on a time scale of tens of milliseconds. The negative *ν*(SiOD) band indicates a temperature increase due to the pump pulse which can be quantified after appropriate calibration, as discussed below.

The recovery of ZOD groups accompanying loss of hydrogen bonded water which is very evident in the FTIR temperature ramp difference spectra in Fig. 3(a) is scarcely seen in the T-jump difference spectra in Fig. 4(a). This difference between FTIR and T-jump spectra is also seen at an initial temperature of 80 °C (ESI Fig. S4†). The missing appearance of free Brønsted sites after heating in the transient data may be an indication that the T-jump causes a large population of Brønsted sites ZOD⋯(OH_2_)_*n*=1,2_ to immediately become deprotonated, with accompanying formation of D^+^(OH_2_)_*n*=1,2_. A minimal free ZOD Brønsted population throughout the heating and cooling process would therefore be expected, as is observed in the data. Since the water present in the sample is deuterated to the same level (10%) as the zeolite acid sites, the amount of D → H exchange from deprotonation of ZOD sites will be matched by H → D exchange of ZO^−^ generated from ZOH sites.

The sample held initially at 175 °C (Fig. 4(b)) contains no adsorbed water clusters (H_2_O_*n*_, *n* > 1). The difference spectra recorded following the T-jump pulse show the negative bands due to loss of hydrogen bonded ZOD groups, the negative SiOD feature caused by the increased temperature and the expected positive feature due to recovery of free ZOD groups, which is distorted by the temperature dependent frequency shift. The relative contributions of desorption and frequency shift to the *ν*(OD) band can be separated by comparing the FTIR and T-jump data at different temperatures (ESI Section 6†). In ESI Section 7† we plot the decay kinetics of the positive (recovery) and negative (frequency shift) components of the *ν*(OD) band for initial sample temperatures of 175 and 300 °C respectively. At 175 °C the two components decay with different time constants. At 300 °C, where no adsorbed water of any kind is present, the only contribution to the difference spectrum is the temperature dependent frequency shift, and the two components decay synchronously.

Examining the spectral evolution of the data in Fig. 4, the negative features in the spectra associated with water desorption increase from a pump–probe delay of *t* = 0 to *ca.* 10^−4^–10^−3^ s, followed by recovery as the sample begins to return to its equilibrium conditions. The shifts of the silanol and free Brønsted ZOD modes due to the heating of the sample evolve on a different timescale to the modes associated with water desorption, evidenced by the water desorption modes persisting in the spectra at 10^−2^ s while these other features are already fully decayed. The kinetics of the silanol and water-associated modes in the spectra at different temperatures are shown in ESI Section 8.† We now turn to establishing the heating and cooling behaviour of the zeolite on laser excitation.

### Heating and cooling of ZSM-5 on laser excitation

**Fig. 5:**
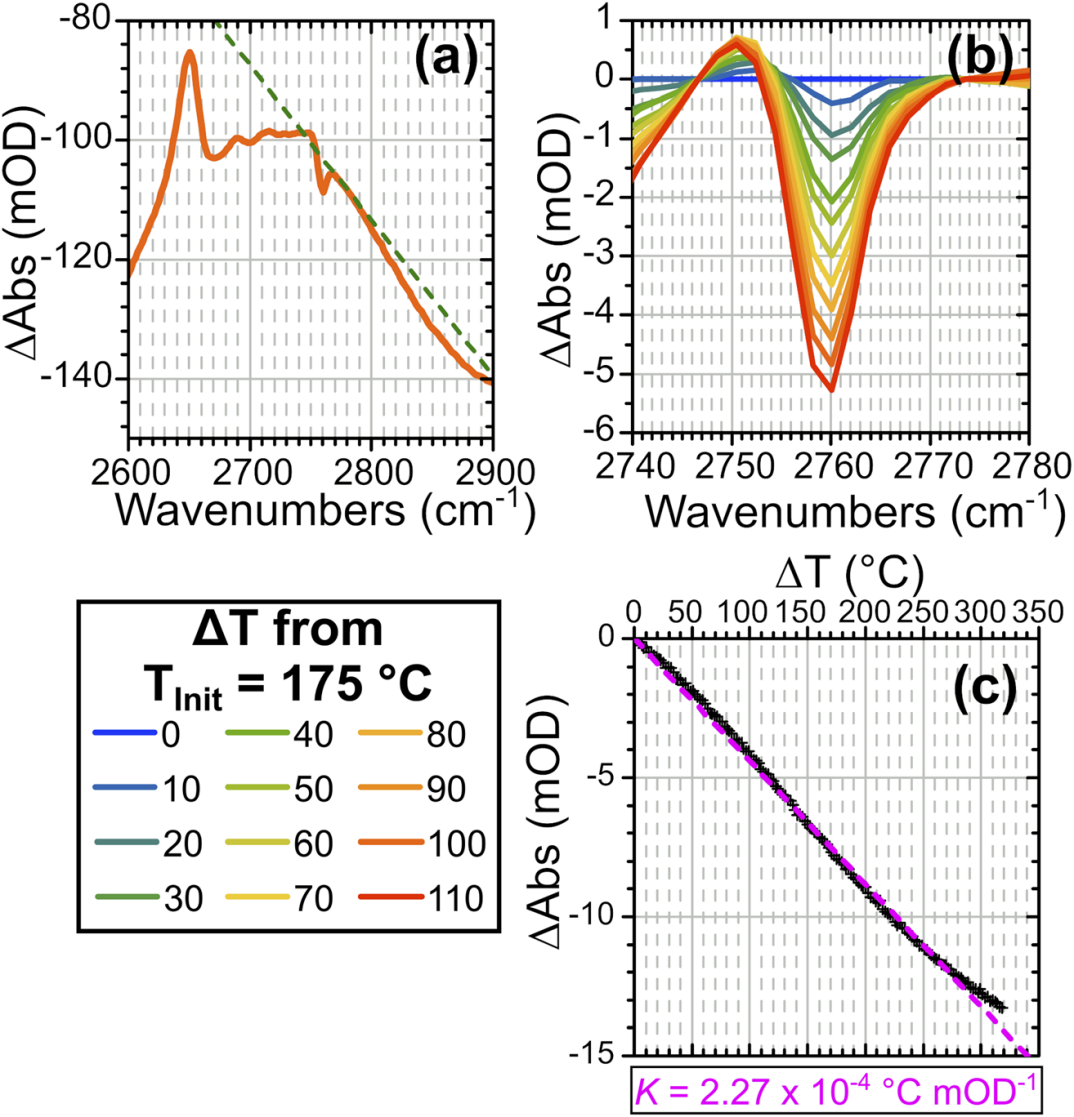
(a) FTIR difference spectrum of Δ*T* = 50 °C from *T*_Init_ = 175 °C showing a linear baseline fit to isolate silanol mode contributions. (b) Comparison of the *ν*(SiOD) feature in baseline subtracted difference spectra at indicated Δ*T* values showing increase in intensity of negative feature with increased Δ*T*. (c) Variation of *ν*(SiOD) negative intensity with Δ*T* showing linear relationship with slope 1/*K*.

To quantify the temperature changes occurring on laser excitation, we explored the effectiveness of the silanol *ν*(SiOD) band as a thermometer. This band shows no interaction with water under the conditions of these experiments, so that variations in its frequency and intensity with temperature provide a way to estimate temperature changes during the *T* jump experiments. Variations in the *ν*(ZOD) band in dry zeolites and the *ν*(CO) bands of trifluoroacetic acid have previously been used successfully as thermometers in other studies in the literature.^14,38^ Fig. S8(a) in the ESI† shows how the silanol band in ZSM-5 varies with temperature in the FTIR spectra. To remove the contributions to the transient spectra from water desorption, baseline subtraction close to the edges of the *ν*(SiOD) band is applied. Applying this also to FT-IR difference spectra from the same starting temperature allows a relationship between the silanol mode absorbance change and increase in temperature to be established. The *ν*(SiOD) band is observed by FT-IR to shift and weaken as a function of increasing temperature. Frequency shifts alone produce a symmetric derivative in the difference spectra, while an absorbance decrease alone would give a negative peak in the difference spectrum. The combination of a frequency shift to lower frequency and an absorbance decrease gives an asymmetric difference band with an enhanced negative lobe on the high frequency side. This is illustrated with simulated spectra in ESI Fig. S9.†Fig. 5 shows how the intensity of this negative lobe in difference FTIR spectra can be used to construct a calibration curve. A linear baseline is fitted to the difference spectrum as illustrated in Fig. 5(a) and the baseline subtracted spectra plotted as a function of the temperature difference in Fig. 5(b). The intensity of the negative lobe is a linear function of the temperature difference (Fig. 5(c)). The linear slope of 1/*K* provides a conversion factor *K* used to convert the intensity of the *ν*(SiOD) difference feature to the corresponding change in sample temperature.

1 Δ*T*(*t*) = *K*ΔAbs_(SiOD)_(*t*)

Values of *K* for initial temperatures between 80 and 300 °C are listed in ESI Table S2.† We confirmed that the calibration curves were independent of the water vapour pressure at least up to 300 °C (see Fig. S10†).

**Fig. 6:**
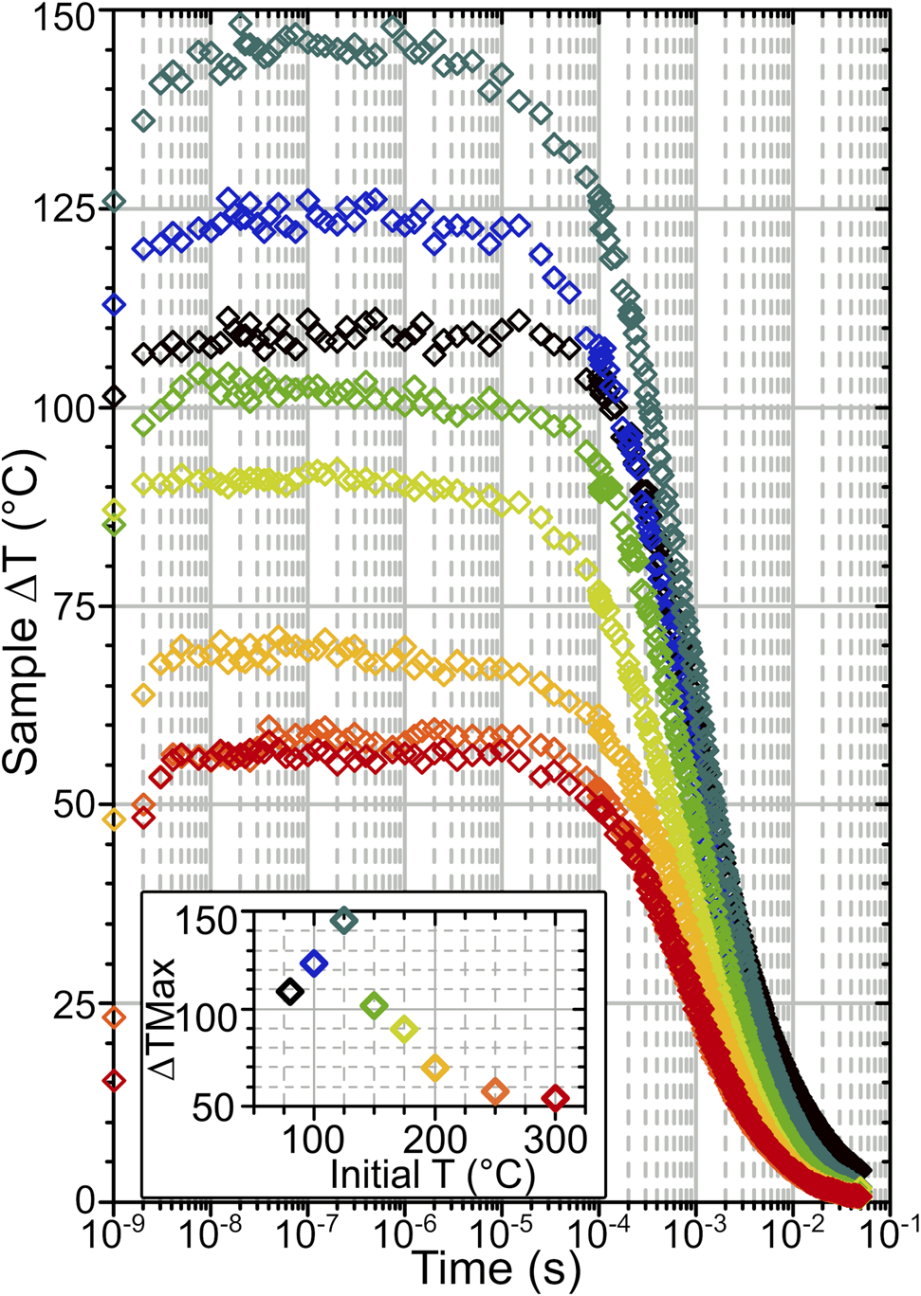
Sample heating *vs.* time after ns laser heating for HOD/H_2_O dosed ZSM-5 at different initial temperatures calculated based on changes in silanol mode intensity. The inset plot shows the maximum Δ*T* achieved against initial temperature. The colour scheme of the heating transients corresponds to that in the inset.

From calibration curves constructed we can then estimate temperature changes occurring in the T-jump experiments. Fig. 6 shows temperature–time dependence curves obtained in this way. The accuracy of the estimate is determined by the fit quality used to derive conversion factor *K* and the degree of noise in the time-resolved spectra, and is ±0.5–3.5 °C for the data presented here. Instantaneous local heating occurs within the 1.5 ns pump pulse duration because vibrationally excited Brønsted sites, water and silanols relax on ∼sub 200 ps timescales.^23,24^ T-jump rise times of ∼4 ns are observed, slightly greater than the measured pulse duration of the pump laser.^14^ This presumably represents the absorbed energy equilibrating from the location of the O–H sites pumped by the laser pulse to the entire framework within each crystallite. Temperatures stabilise after 3–10 × 10^−9^ s, after which the zeolite remains hot out to the millisecond time range. The magnitude of the T-jump achieved (Δ*T*_Max_) varies depending on starting temperature (Fig. 6 inset). This is due to the varying levels of water in the sample changing the absorbance in the pump region of the spectrum and therefore the amount of energy absorbed from the pump pulse. The source of this effect is demonstrated by the fact that the samples at 250 and 300 °C are both free of adsorbed water and therefore have virtually identical IR spectra (Fig. 2(a) and S4†) and T-jump magnitudes (Fig. 6 inset). Δ*T*_Max_ peaks at a *T*_Init_ value of 125 °C, below which Δ*T*_Max_ decreases, despite the pump absorbance of the sample continuing to increase. The source of this behaviour will be considered in more detail below.

The cooling behaviour of the samples was analysed by fitting the temperature curves of Fig. 6. Points earlier than 10^−7^ s were excluded from the fit in order to capture just the cooling. In all cases the data was found to be best represented by a bi-exponential function of the form:2
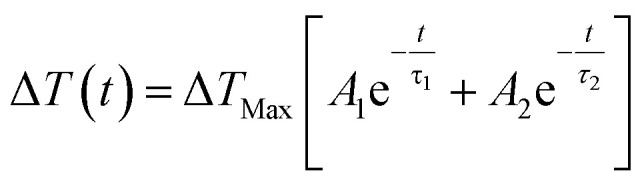
*τ* is the time constant for each decay term, Δ*T*_Max_ the maximum Δ*T* achieved and *A* the relative contribution of each term to the overall cooling curve. The fit parameters for each temperature are given in the ESI, Section 10.† The first exponential fit to a time constant of *ca.* 0.7 ms accounted for the majority (∼70%) of the cooling in each case. The second term, still significant, represents a slower cooling process (time constant *ca.* 6.4 ms). The samples at 80 and 100 °C exhibit deviations from the trends observed at higher starting temperatures, with slower cooling and larger values of *τ*. To explain why the cooling is bi-exponential, in the next section we examine the temperature distribution within the laser-pumped zeolite sample.

## Heat transfer simulations in laser pumped zeolites

### Simulation method

To understand the time dependent temperature distribution within the ZSM-5 pellets during and following the T-jump laser pulses, the spatial temperature profile and its evolution over time was simulated in three dimensions. Assuming radial symmetry about the excitation laser pulse centre, the model was based on the 2D axisymmetric heat equation in cylindrical coordinates solved numerically using finite element analysis in Mathematica Version 13.2.^39^ The model was solved with logarithmic time steps, to allow high time resolution immediately after the laser pulse, while still following the cooling profile out as far as 1 second. Further details are provided in the ESI, Section 11.†

The determination of the simulation input parameters, namely the pump pulse energy, pump and probe spot size, sample absorbance, pellet thickness, sample density, zeolite specific heat capacity (*C*_P_) and thermal conductivity (*κ*), pump beam quality factor and reflection coefficient, are discussed in ESI Section 12.† The effect of the zeolite heat capacity was found to be particularly significant in determining the magnitude of the temperature jump achieved and was modelled using a second-order polynomial derived from fitting experimental data from Lu *et al.*^40^ The effect of pump light scattering from the pellet, heat transfer through convection or radiation and the potential temperature-dependence of parameters other than *C*_p_ over the course of the simulation run were not included due to difficulties in quantifying their effects. The effects of asymmetric beam profiles, pump–probe alignment, and the variation of the probed laser spot within the base temperature profile of the zeolite pellet are assumed negligible. These factors are examined in more detail in the ESI, Section 13.† Their net effect is that simulation Δ*T* values are likely to be slightly larger than those observed experimentally, as they represent a more ‘ideal’ set of conditions.

The output of the simulations are time dependent 2D distributions of temperatures, whereas the temperatures characterised from experimental data represent an average temperature across the probed region. In order to allow comparison between these different types of data, the simulation outputs were converted to a single spatially-averaged temperature for each time point weighted to account for the variation in probe intensity across the probed region (ESI, Section 14†). Finally, to validate the entire model, T-jumps in water sandwiched between CaF_2_ windows, and in free-standing mordenite pellets, were simulated and compared with experiments/simulations reported in the literature^14,15,23^ (ESI, Sections 15 and 16†).

### Simulated temperature jumps

**Fig. 7:**
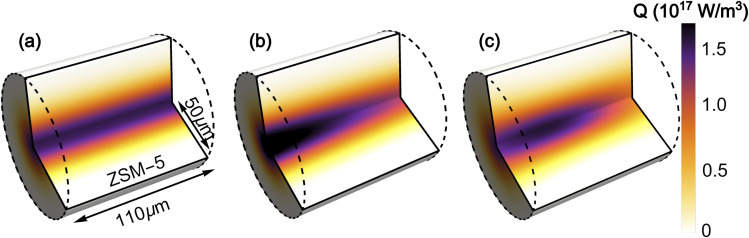
3D distribution of heat supplied by the pump laser in heat transfer simulations showing in sequence (a) a Gaussian pump beam passing unattenuated through the sample (b) the added effect of attenuation of the pump as it passes through the sample (c) added effect of the divergence of the pump beam.

The simulated laser pulse heating results in an inhomogeneous temperature distribution in both dimensions of the ZSM-5 pellet (Fig. 7). The radial temperature inhomogeneity is Gaussian, due to the Gaussian profile of the pump laser beam. Temperature inhomogeneity along *z* is due to a combination of the absorption of the pump beam as it travels through the sample (resulting in an exponential decay in the amount of energy delivered) and the divergence of the tightly focussed pump beam (resulting in lower intensity at the faces of the pellet than at the pump focus). The highest temperatures in the sample are located in the first half of the pellet depth (see ESI, Section 17†).

Cooling of the sample post-pumping occurred primarily through radial conduction into the unpumped region of the zeolite pellet. Examination of the effects of cooling into the surrounding air (ESI Section 19†) found the contribution of these processes to be minimal. The dominating parameters affecting the rate of radial cooling within the simulation were found to be the pump and probe spot sizes and the thermal conductivity of the zeolite, *κ*. While the spot size was well characterised by experimental measurements (ESI Section 3†) it was not possible to determine a reasonable estimate for the value of *κ* from the literature. A range of values were determined empirically by finding the range of *κ* values for which the fitted time constants fell within the confidence intervals of the experimental cooling curve, with each of the other simulation parameters allowed to vary within their own confidence intervals. This gave a value of *κ* = 0.25 ± 0.1 W m^−1^ K^−1^ for simulations with *T*_Init_ from 125–300 °C. The predicted value of *κ* increases with temperature which is consistent with the results obtained for measurements of thin films,^41–43^ although the *κ* values determined from our experiments are approximately 4 times smaller, possibly indicating the much lower level of thermal contact between crystallites than in a pure film. It was not possible to derive a value for the thermal conductivity that produced cooling behaviour consistent with the experimental observations at 80 and 100 °C, indicating that at these temperatures there are additional effects from adsorbed water that the simulations do not account for. This results in a deviation of the experimental and simulated T-jump results at low temperatures, which is considered in more detail below. The derived values for *κ* are given in Table S3 in the ESI (Section 10).† The analysis approximates the thermal conductivity as constant during the heating and cooling process.

## Comparing simulation and experiment

Simulated temperature–time curves of the laser heated zeolites were generated using the best-estimate values for the simulation input parameters at each temperature. Additional simulations were carried out to establish the maximum and minimum T-jumps possible within the degree of uncertainty in the input parameters, and these were used to generate the error bars of the reported simulation results.

**Fig. 8:**
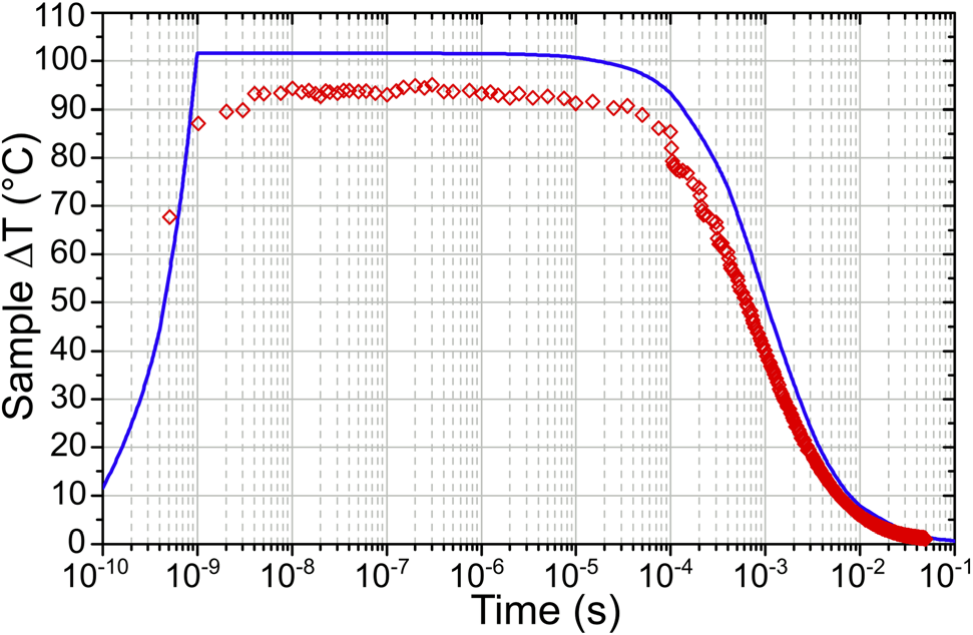
Average sample temperature across the probed region *vs.* time for a temperature jump from 175 °C *T*_Act_ as determined experimentally (red) and from simulations (blue). The simulation uses the best estimates for input parameters.

Fig. 8 shows both the simulated and experimentally derived temperature profiles for a T-jump from 175 °C *T*_Act_ as an example. It can be seen that the experimental results correspond closely with those derived from the simulation, with the maximum experimental Δ*T* of 89 °C being 85% of that predicted by the simulation. The uncertainties in the input parameters gave an error bar in the simulated Δ*T* of ±40 °C, however the close agreement with the experimental results indicates that the best-estimate input parameters are realistic.

**Fig. 9:**
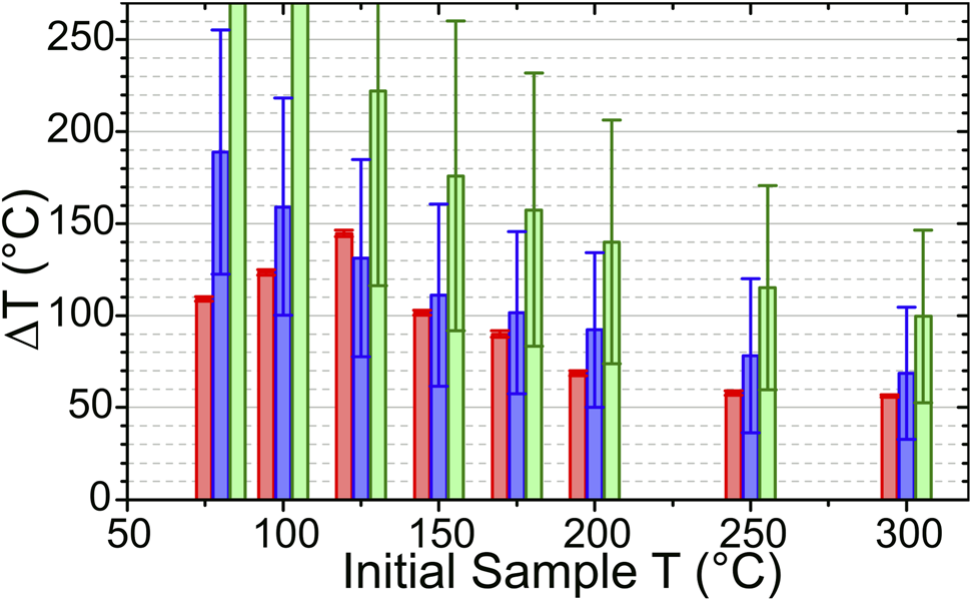
Average temperature jump measured between 10^−8^ and 10^−5^ s as determined by: experiment based on changes in silanol mode intensity (red); heat transfer simulations (blue); energy absorption calculations (green). Error bars for simulation and calculation values are based on the uncertainties in the input parameters.

In the simulations, increasing *T*_Init_ had the effect of increasing the zeolite specific heat capacity and removing water from the sample, decreasing the sample absorbance in the laser-pumped OH stretch region of 3600 cm^−1^. These changes result in the simulated T-jump magnitude diminishing with increased starting temperature. Fig. 9 compares the T-jump magnitude, Δ*T*, achieved at each temperature in both the experimental and simulated datasets. The reduction in simulated and experimental Δ*T* with increased temperature is readily apparent. It can be concluded that the variation in pump absorbance of the zeolite sample has the greatest effect on T-jump magnitude Δ*T* variation with start temperature, with changes in heat capacity being small enough in this temperature range that they are of secondary importance.

As an alternative to the simulations described above, a simple way of computing Δ*T* is to simply consider the amount of energy *E*_pump_ absorbed by the sample of density *ρ* and optical density OD in a volume (*V*) defined by the beam waist and pathlength:^44^3
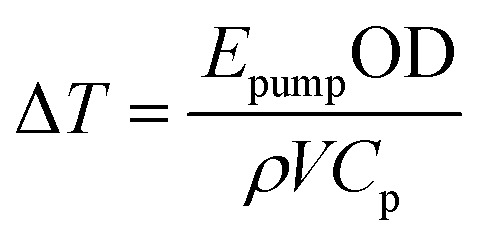


ESI, Section 18† describes the estimation of heated volume, *V*. Temperature jumps computed from eqn (3) are shown in Fig. 9 and overestimate the value of Δ*T* at all temperatures, reflective of the fact that this represents the maximum achievable T-jump magnitude without considering the weighting of the measured temperature due to the profile of the probe beam, inhomogeneous distribution of the pump beam energy, or the effect of evolution of the sample heat capacity over the course of the laser heating cycle.

**Fig. 10:**
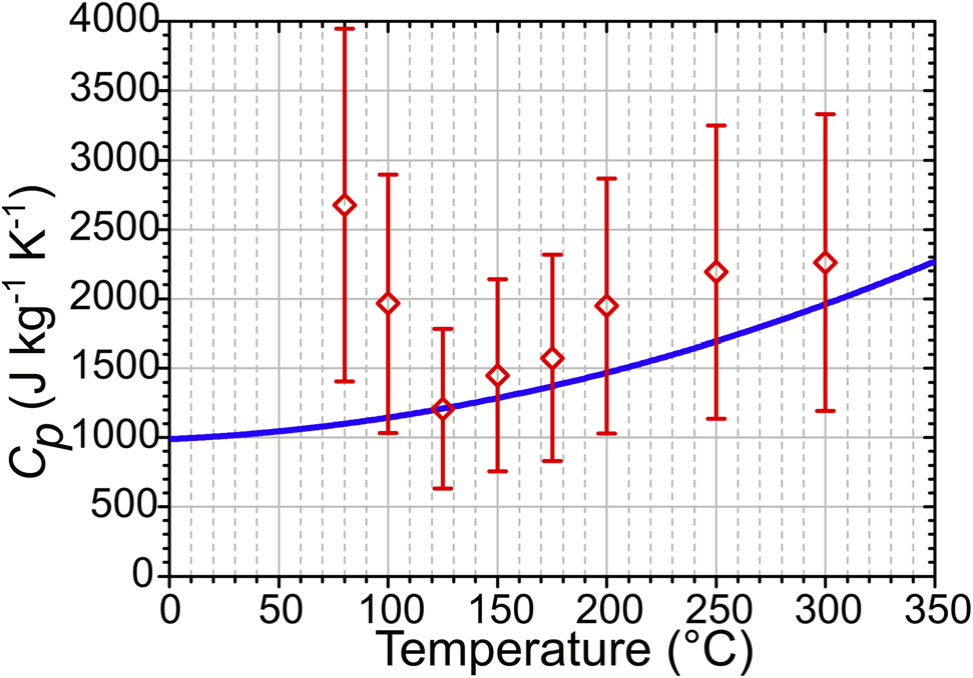
Zeolite *C*_p_ values calculated from experimental T-jump data using eqn (3) (red) compared to simulation input *C*_p_ values derived from ref. 40 (blue).

Water uptake *via* the formation of water clusters in ZSM-5 at temperatures below 125 °C will increase the heat capacity of the zeolite sample. This is not accounted for in either set of simulated Δ*T*_Max_ values, which continue to increase with decreasing temperature down to the lowest temperature measured. In contrast, the experimental temperature jump magnitudes diminish at these lower temperatures. An estimate of the zeolite heat capacity (*C*_p_) from experimental Δ*T* values can be obtained through use of eqn (3) (with details of this calculation given in ESI Section 18†). These estimated *C*_p_ values match those from Lu *et al.*^40^ used in the simulations at *T* ≥ 125 °C (Fig. 10). At lower temperatures, these estimates are up to 250% of the value predicted for the zeolite alone, indicating that water clusters within the zeolite raise the heat capacity of the sample. That the difference in predicted and experimentally-estimated *C*_p_ values is so large suggests that this difference in heat capacities is likely to be responsible for the majority of the deviation from simulated heating behaviour observed experimentally below 125 °C.

We now return to the bi-exponential cooling of the zeolite samples observed experimentally. The simulated sample cooling was also found to be bi-exponential regardless of the value of thermal conductivity used, with only the magnitude of the time constants varying with *κ*. Fit parameters are reported in the ESI, Section 10.† The *τ*_1_ : *τ*_2_ ratio and *A*_1_/*A*_2_ for a given start temperature were the same, whether averaged over the FWHM diameter of the probe beam, or at different single-positions in the sample. The simulations therefore indicate that the bi-exponential cooling is not a consequence of averaging the temperature over the probed region of the sample. Although both cooling processes are slowed by the presence of water clusters, *τ*_2_ is more strongly affected, leading to the *τ*_2_/*τ*_1_ ratio being proportional to the level of water clusters at low temperatures. The neglect of the properties of water from the simulation explains the inability to match the simulated cooling to experimental results at temperatures ≤125 °C.

By carrying out a series of laser heating simulations of different heat distributions within the zeolite (described in the ESI, Section 19†), the bi-exponential nature of the sample cooling was found to be phenomenologically due to redistribution of the inhomogeneous thermal energy profile within the heated region of the zeolite. Newton's single exponential law of cooling assumes that heat redistribution within the heated region is much faster than the overall cooling, either due to the volume of the heated region being insignificant or the thermal conductivity (*κ*) of the heated region being higher than the surroundings into which it cools.^45–48^ The laser-pumped sample volume is large enough for an inhomogeneous heat distribution to be present immediately post-pumping. Cooling occurs primarily through conduction into the surrounding, cooler zeolite, whose *κ* value differs minimally from that in the heated region. Redistribution of the thermal profile in the heated region and cooling into the unheated zeolite therefore occur on similar timescales resulting in bi-exponential cooling. Increasing the degree of inhomogeneity in the sample heating has the effect of increasing the relative contribution of the ‘slow’ *τ*_2_ term to the overall cooling curve. In the experimental data, the inhomogeneous temperature distribution arises from the Gaussian, divergent nature of the pump beam and the ratio of *κ* between the pumped and unpumped regions. The relative contribution of the *τ*_2_ term is essentially constant for all *T*_Init_ values (*A*_2_ = 0.33 ± 0.02). This implies that in the bi-exponential cooling, the ‘fast’ term represents the overall cooling while the ‘slow’ term represents the thermal energy redistribution.

## Multiple-pulse heating

**Fig. 11:**
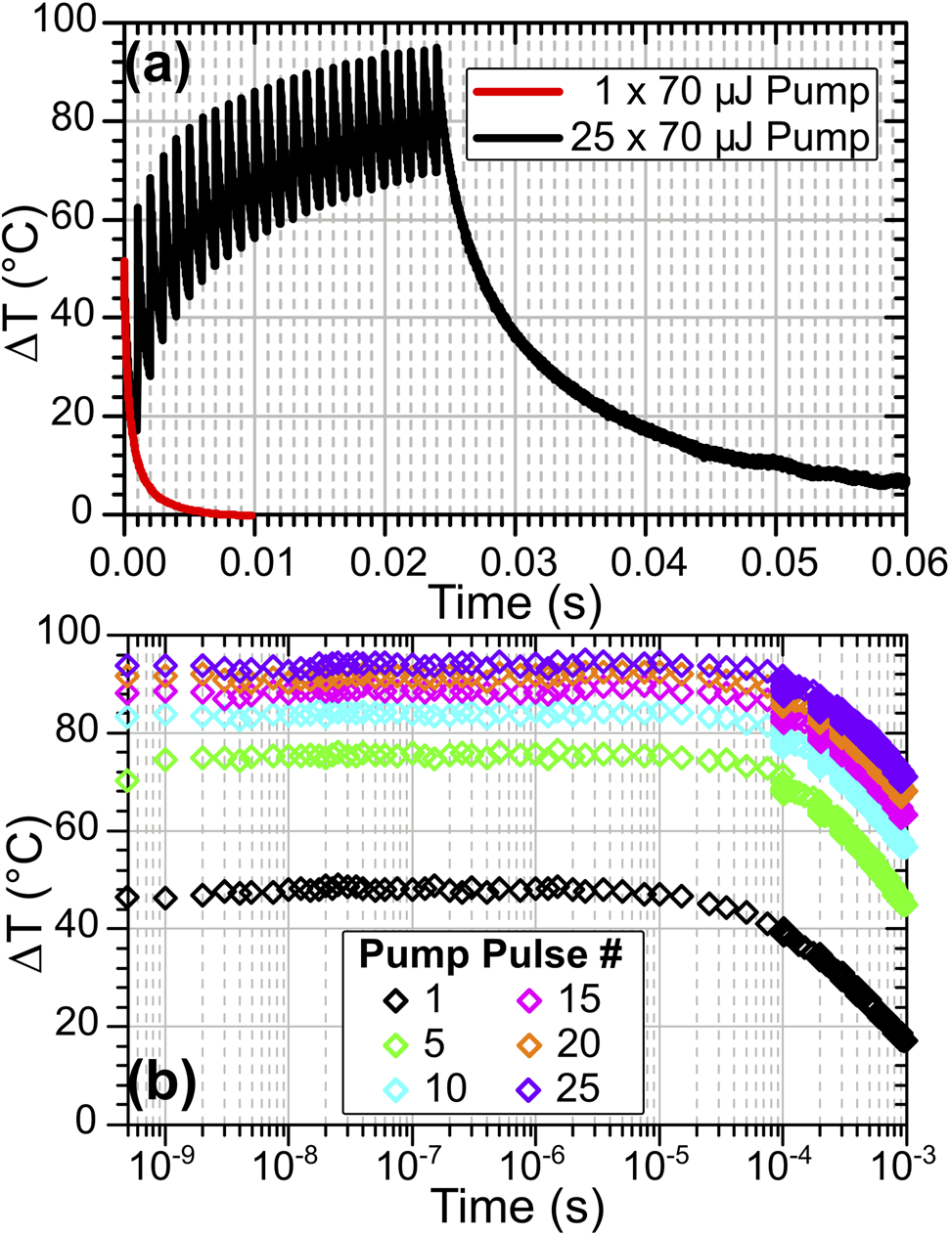
(a) Sample temperature evolution for HOD/H_2_O in ZSM-5 at *T*_Init_ = 125 °C following pumping by 1× (red) and 25× (black) 70 μJ pump pulses. (b) Comparison of temperature evolution in first 1 ms following indicated pulse number in 25-pulse burst showing nanosecond time resolution throughout full range.

While the results reported above show that significant T-jumps are achievable with a single pump pulse, even larger heating magnitudes are possible by changing the pump trigger to initiate a variable-length burst of pump pulses at 1 kHz, pumping the sample repeatedly in quick succession. This approach has similarities to the modulated CW laser heating method reported for solution studies by Ashwood, *et al.*,^15^ but retains nanosecond time resolution and direct excitation of fundamental modes. Fig. 11 compares the sample heating in a single-pulse heating experiment on HOD in ZSM-5 at *T*_Init_ = 125 °C with the same sample pumped with a 25-pulse burst. This experiment was performed with the pump OPO set at a wavelength of 2.9 μm rather than the 2.8 μm used in the results reported above, an area of the sample IR spectrum with lower absorbance, explaining the smaller single-pulse T-jumps achieved compared with Fig. 6.

As shown in Fig. 11(a) for 1 kHz burst pumping of ZSM-5, the second and subsequent pulses arrive before the sample fully cools, meaning that each subsequent pulse heats the sample to higher temperatures. Over the sequence of pump pulses, although the heating reduces as water is desorbed from the sample (which reduces the pump absorbance), heating continues throughout the burst. The rate of temperature gain per pump pulse becomes constant once the sample reaches a fully-desorbed state at *ca.* 200 °C, which is after pulse # 10 (Δ*T* ∼80 °C). This means that arbitrarily large T-jumps may be achieved in zeolite samples through use of a sufficiently long pump burst, subject to the maximum repetition rate of the pump laser. Computer simulations indicate that for the setup in Fig. 1, T-jumps as large as 350 °C above starting temperature can be achieved with a 50-pulse burst, as shown in the ESI, Section 20.†

Use of TRMPS probing means that it is possible to observe each individual T-jump in the pump burst with nanosecond time resolution, allowing the evolution of the sample to be observed at each successive temperature caused by each laser pulse of the burst (Fig. 11(b)). Fitting of the sample cooling kinetics for each pulse shows the fast bi-exponential decay constant increasing from *ca.* 1 to *ca.* 3 ms over the course of the burst process. This can be attributed to the heated volume of the sample enlarging with the repeated laser pulses relative to the laser-pumped volume. Simulations indicate that the full-width at half maximum of the radial temperature distribution increases from 36 μm after a single pulse to *ca.* 80 μm after a 50-pulse burst (ESI, Section 20†). The subsequent reduction in thermal gradient in the radial direction leads to slower cooling.

## Conclusions

Pulsed-laser heating with time-resolved multiple probe infrared absorption spectroscopy and tightly focussed pump and probe beams has been applied to induce and study T-jumps in nanoporous zeolite catalysts. The single-shot temperature jumps achieved are 5× larger than those previously described for time-resolved heating studies in solution^14,15^ and enable the possibility of using pulsed laser heating to selectively activate and study heterogeneous catalytic activity in reactive systems.

The magnitude of T-jumps in partially hydrated zeolite systems were measured through the use of temperature-related changes in the positioning of the *ν*(SiOD) silanol mode. This allows us to determine that our approach is able to produce T-jumps of ≥50 °C from a pump energy input of 70 μJ in the bare zeolite. Even larger temperature changes are achievable at temperatures where the sample contains adsorbed water, increasing absorption of the pump laser light. Use of multiple-pulse pumping allows temperature jumps to be extended to achieve arbitrarily large increases in temperature while still providing nanosecond-resolution information on the sample after each pulse of the burst.

The rate of cooling of the ZSM-5 sample studied was unaffected by starting temperature in cases where the sample contained ≤1 water molecule per acid site and resulted in the sample remaining at least 10 °C above base temperature for >1 ms in all cases. Owing to the increased presence of water clusters, low starting temperatures show decreased T-jump magnitude and slower recovery. One particular feature of interest is the weaker than expected appearance of free Brønsted site in the transient infrared data upon loss of hydrogen bonded Brønsted site. Further tests will be required to determine whether this is due to laser induced deprotonation of the Brønsted site, as suggested by the data.

Using fundamental zeolite properties, laser beam properties and IR absorption of the sample, two-dimensional heat transfer modelling accurately describes the experimental results with respect to both Δ*T* and bi-exponential cooling rate, whilst also providing information on the heat distribution within the sample. The close agreement with experimental results supports its use as an approach for investigating T-jumps in zeolite/adsorbate systems which do not have convenient modes for spectroscopic determination of the magnitude of Δ*T*. Simulated Δ*T* values for such systems can be calibrated using ZSM-5 as a reference system to derive a correction factor accounting for parameters difficult to determine accurately, such as pump–probe overlap.

The method of T-jump spectroscopy described here can be applied to other zeolite materials, provided that they have suitable optical density and thickness. Use of the silanol thermometer method to evaluate the temperature changes in the sample requires that any adsorbates do not interact strongly with the silanol group, as this will disturb the linear dependence of the *ν*(SiOD) mode on sample temperature. For a given zeolite, the Δ*T* achieved and cooling rate of the excited sample varies according to the *C*_p_, *κ* and pump absorbance. These are the primary properties influencing the T-jump behaviour. Reported *C*_p_ and *κ* values of different zeolites have been observed to vary by up to ±70%,^40,49^ indicating that single pulse laser heating of 35 to 210 °C is possible using the approach reported here.

## Data availability

Data available on reasonable request.

## Author contributions

Alexander Hawkins (investigation, writing – original draft), Amy Edmeades (investigation, software, writing – original draft), Christopher Hutchison (investigation), Michael Towrie (investigation, writing – review and editing), Russell Howe (investigation, writing – review and editing), Greg Greetham (investigation, writing – review and editing), Paul Donaldson (investigation, writing – review and editing, project administration, funding acquisition).

## Conflicts of interest

There are no conflicts to declare.

## Supplementary Material

SC-015-D3SC06128K-s001
