# Supplementary material for: Laser induced temperature-jump time resolved IR spectroscopy of zeolites
Source: Chem Sci. 2024 Feb 7;15(10):3453–65. doi: 10.1039/d3sc06128k (PMC10915812; doi:10.1039/d3sc06128k)
Supplement: SC-015-D3SC06128K-s001 [file SC-015-D3SC06128K-s001.pdf]

Electronic Supplementary Material (ESI) for Chemical Science.
This journal is © The Royal Society of Chemistry 2024

Laser induced temperature-jump time resolved IR spectroscopy of zeolites – Electronic Supplementary Information

Alexander P. Hawkins,[a] Amy E. Edmeades,[a] Christopher D.M. Hutchison,[a] Michael Towrie,[a] Russell F. Howe,[b] Gregory M. Greetham,[a] Paul M. Donaldson,*[a]

[a] *Central Laser Facility, Research Complex at Harwell, STFC Rutherford Appleton Laboratory, Didcot, Oxon, OX11 0QX, UK.*

[b] *Department of Chemistry, University of Aberdeen, Aberdeen, AB24 3UE, UK.*

Table of Contents

S1. Gas Flow Apparatus: 3

S2. Timing and Sample Movement Arrangements for Time-Resolved Spectroscopy 3

S3. Pump and Probe Beam Profile Measurements 5

S4. Additional FT-IR Difference Spectra of HOD in ZSM-5 6

S5. Time-Resolved IR Data at Additional Temperatures 7

S6. Direct Comparisons of FT-IR and Time-Resolved Data 8

S7. Deuterated Brønsted Kinetics Providing Evidence for Two Processes at 175 °C 9

S8. Comparison of Silanol and H-Bonded Brønsted Decay Kinetics at Different Temperatures 10

S9. Deriving Temperature Change Values from Time-Resolved IR Data 11

S10. Fit Parameters for Cooling Curves 14

S11. Equations Used for Zeolite Heat Transfer Simulations 16

S12. Simulation Input Parameters 18

12.1 Zeolite Thermal properties 18

12.2 Zeolite Optical Properties 20

12.3 Pump laser parameters 21

S13. Experimental Factors Excluded from Simulation Model 22

S14. Obtaining temperature-time curves from simulations by spatial averaging 24

S15. Comparing Simulations of Water Heating with Literature Data 24

S16. Comparing Simulations of Zeolite Heating with Literature Data 28

S17. Divergence of Pump Beam 28

S18. Deriving Heat Capacity from Experimental Data 29

S19. Investigating the Origins of Biexponential Cooling 30

S20. Computer Simulations of Multi-Pump Heating 33

References 35

S1. Gas Flow Apparatus:

Figure S1 shows the gas handling apparatus used for purging, deuteration and HOD/H_2O loading of the ZSM-5 samples for spectroscopy. All tubing was 1/8" outside diameter stainless steel connected using compression fittings (Swagelok Inc.).

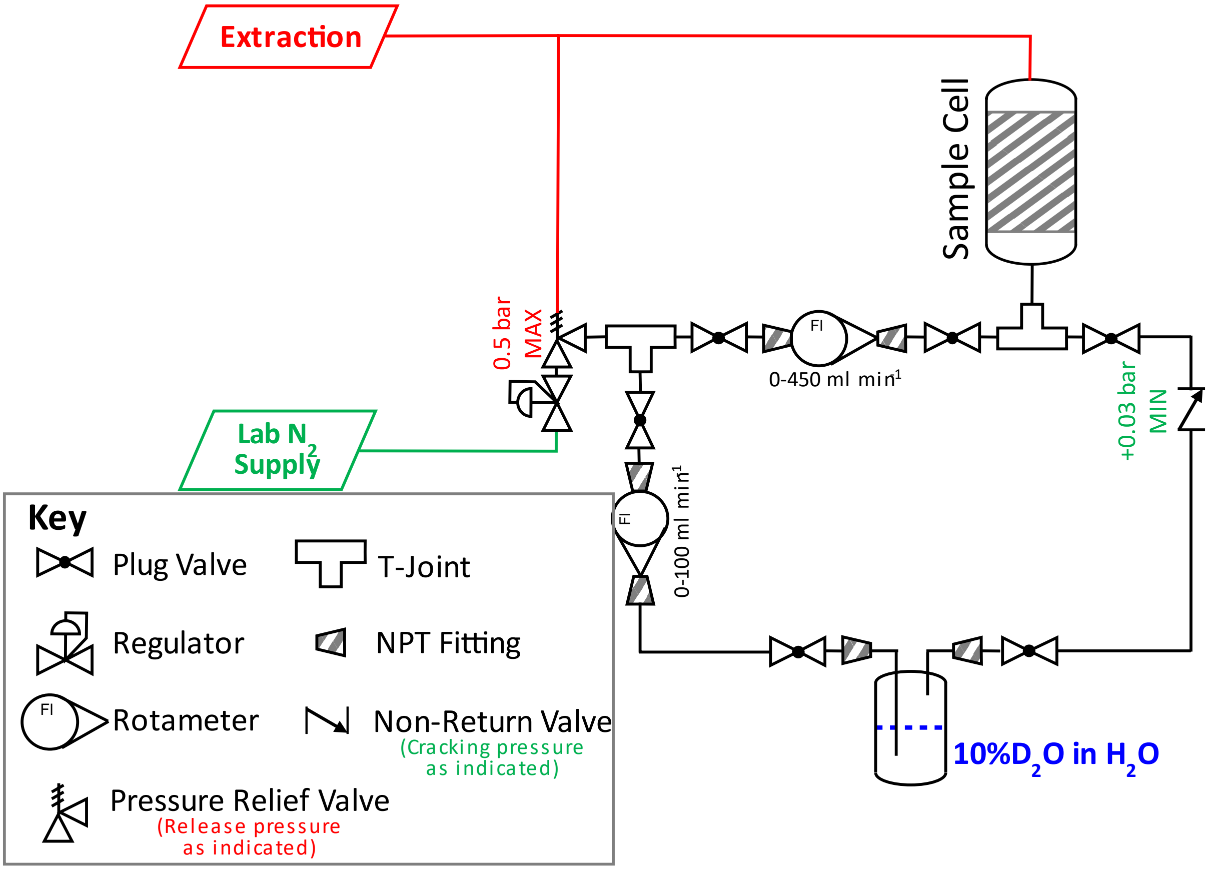

Figure S1: Schematic of gas handling apparatus used for spectroscopy experiments.

S2. Timing and Sample Movement Arrangements for Time-Resolved Spectroscopy

The probe and pump laser systems for time-resolved spectroscopy measurements are described in the main text. To synchronise their operation, the 10 kHz probe clock was divided down to twice the desired measurement rate (20 Hz / 8 Hz) using a computer controlled electronic pulse delay generator (DG#1, Stanford DG645). The signal from DG#1 was used as the external trigger for two further delay generators, DG#2 (Stanford DG645) and DG#3 (Stanford DG535).

DG#2 controlled the pump laser, introducing a delay of 802 µs (measured as the time zero of the probe system) ±0-75 ns, with the variability set by the data acquisition system to collect spectra with sub-10 µs time resolution by shifting the probe pulses relative to the pump pulse. The delayed signal provided a synchronized 1 kHz signal to the laser diode and triggered the Nd:YAG Q-switch matched to the trigger signal. When performing multiple-pump measurements, a series of 2 – 50 signals to the Q-switch at 1 ms intervals was generated using the pulse generator's internal burst generation capability.

DG#3 used a delay longer than the timing signal interval to induce a rate error and generate a signal on every second pump pulse. This was supplied to the data acquisition system, to correctly identify the pump on and pump off measurements, and provided synchronisation for the optical chopper which was used to block every second pump. In the case of 4 Hz measurements, DG#3 also provided a signal at 60 ms delay to trigger sample stage movement.

The sample movement stage consisted of two perpendicularly mounted linear translation stages (Newport XMS-50) controlled by a motion controller (Newport XPS-D8) providing up to 50 mm of motion in the x- and y-axes relative to the beam path and synchronised to each pump laser pulse. This was used to move the sample cell in 200 µm steps in a serpentine pattern across a 3 × 1 mm area of the zeolite sample, triggered by a signal from DG#3 and ensuring that each measurement started from an unpumped region of the zeolite. The sample movement and stage-settling oscillations were visible as additional signal in the time-resolved spectrum (Figure S2). The delay in the trigger signal was optimised to allow the maximum amount of sample decay to be recorded while still completing all stage movement before the end of the data collection period. To ensure that the pump chopper and sample movement triggers were in phase a test measurement was taken for each experiment where it was checked that the recorded signal returned to zero after the stage movement.

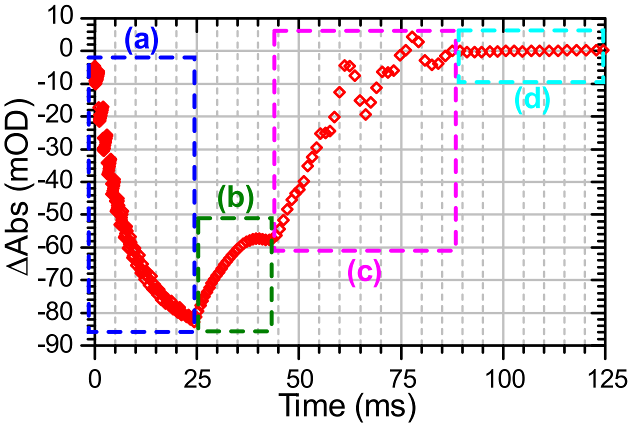

Figure S2: Time-resolved IR spectral intensity at 2750 cm^{-1} for a sample of HOD/H_2O in ZSM-5 excited with 25 × 70 µJ pump pulses showing changes due to pump excitation of the sample (a), sample cooling (b), noise from stage movement (c) and a return to the unpumped state post-movement (d).

S3. Pump and Probe Beam Profile Measurements

To characterise the pump and probe beams, each was imaged at the focus of the aspheric zinc selenide condenser lens of the spectrometer with a microbolometer array (Seek Thermal Compact, pixel pitch 12 µm, modified by removing imaging lens and extruding lens mount). Cuts across the intensity maps of the beams along the major and minor axes were fitted to Gaussians, with the results given in Table S1. Additional measurements were taken 50 and 100 µm from the focus in the *z*-direction in order to measure the divergence of each beam. The average FWHM in each case was used as the spot size for simulation and analysis purposes. Having the probe beam smaller than the pump reduced the variation in temperature across the probed region, although it did not eliminate this effect entirely.

The pump laser power output was measured at 1 kHz repetition rate with a Thorlabs PM160T power meter located in place of the sample at the beam focus and converted to give the average pump energy of 71 ±5 µJ per pulse quoted in the main text.

Table S1: Beam profile parameters taken from fitting of microbolometer data, with 95% confidence intervals.

	Pump Beam (µm)	Probe Beam (µm)
**FWHM (Major Axis)**	36.9 ± 1.9	24.8 ± 0.6
**FWHM (Minor Axis)**	35.0 ± 0.9	18.1 ± 1.3
**FWHM (Average)**	36.0 ± 1.4	21.5 ± 1.0

S4. Additional FT-IR Difference Spectra of HOD in ZSM-5

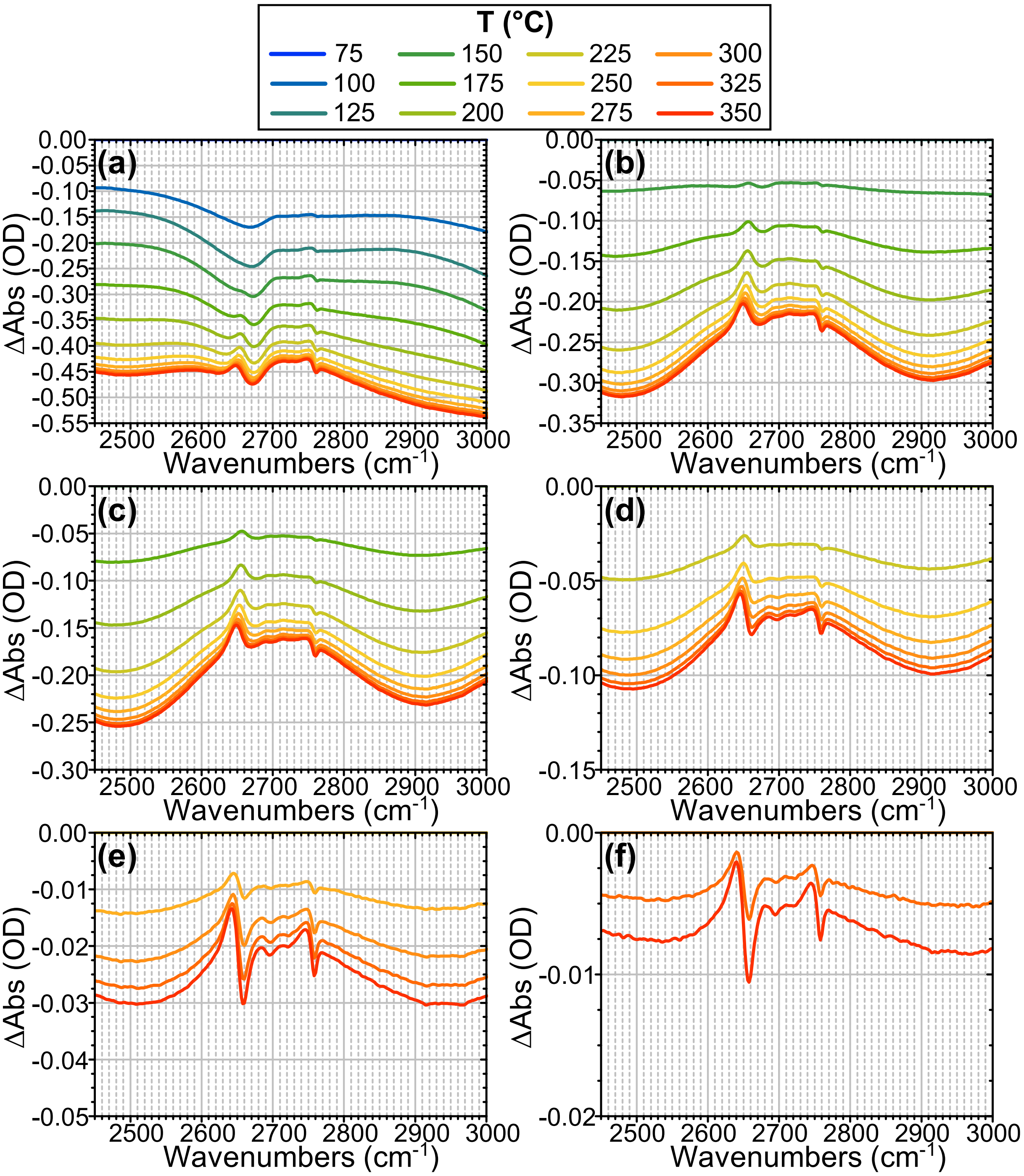

Figure S3: Difference FT-IR spectra on heating at the indicated temperature relative to (a) 75 °C, (b) 125 °C, (c) 150 °C, (d) 200 °C, (e) 250 °C, (f) 300 °C.

S5. Time-Resolved IR Data at Additional Temperatures

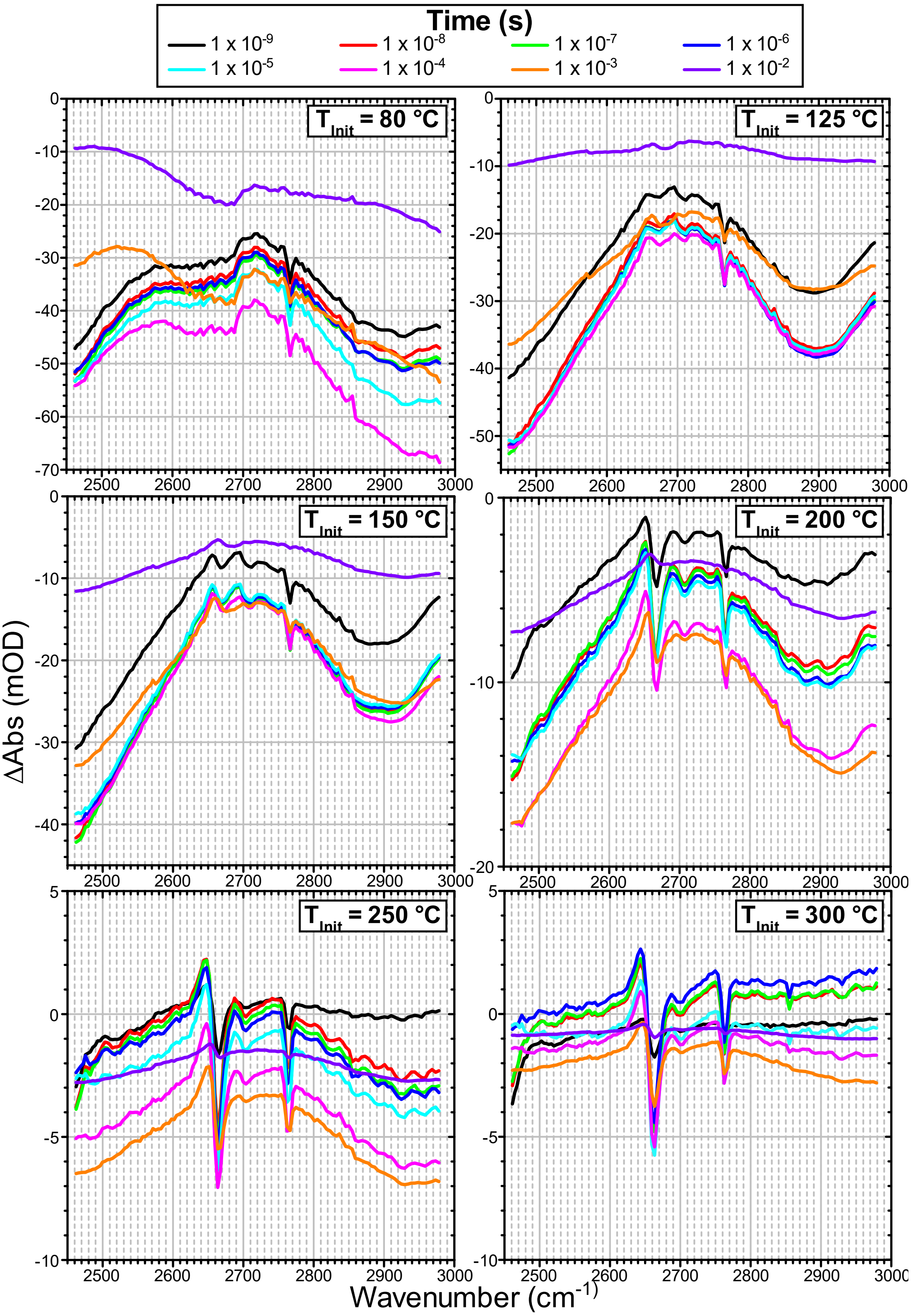

Figure S4: Time-resolved IR spectral evolution of HOD/H_2O vapour-dosed ZSM-5 at indicated T_{Init} values. Spectra are shown at logarithmic time intervals from 10^{-9} – 10^{-2} s following pumping with the 70 μJ laser pulse.

Figure S4 shows the time-resolved IR data equivalent to Figure 4 in the main text for the other 6 values of T_{Init} measured, showing the evolution of sample behaviour as it progresses from a fully HOD/H_2O loaded condition at 80 °C to the dry zeolite spectrum at ≥ 250 °C.

S6. Direct Comparisons of FT-IR and Time-Resolved Data

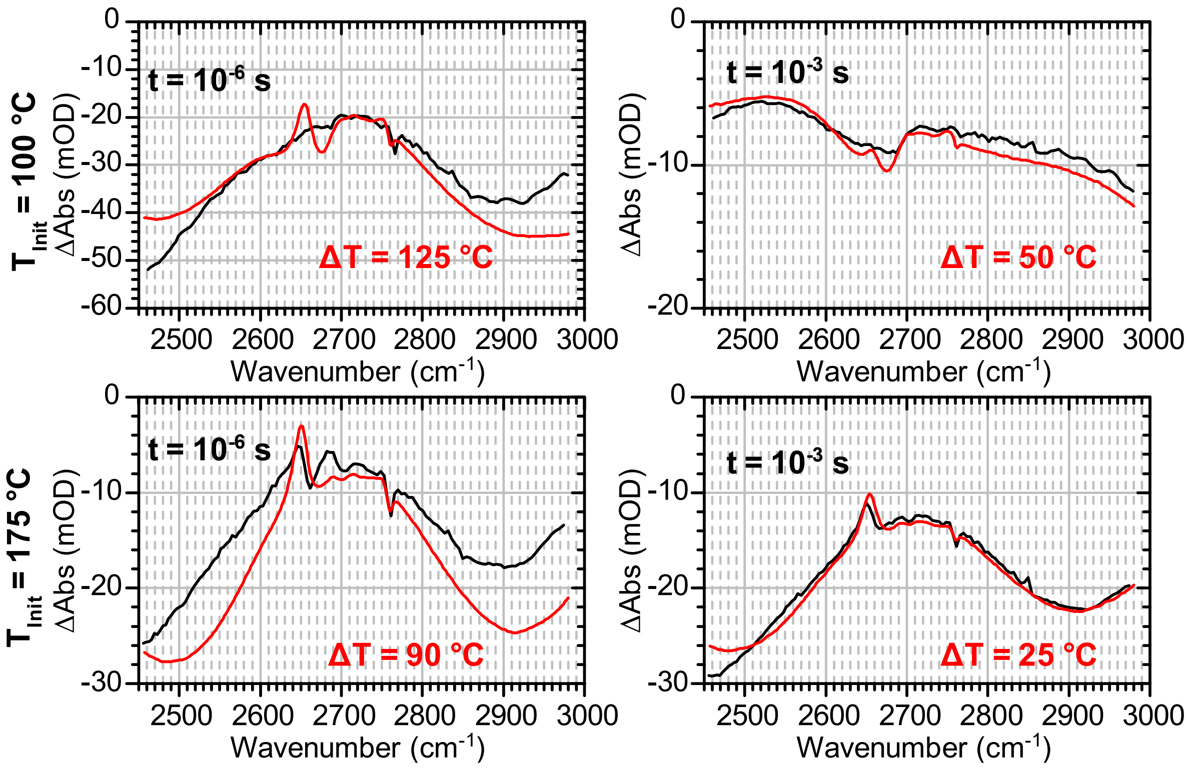

Figure S5: Direct comparison of time-resolved IR spectra at indicated times post-pumping (black) with the FT-IR difference spectra on heating to the indicated ΔT (red) for samples with initial temperatures of 100 °C (top) and 175 °C (bottom). FT-IR spectra have been scaled to account for differences in sample pellet thickness between the two kinds of measurements and shifted in *y* axis to correct for the effects of the water desorption not reaching baseline to enable easier comparison of the modes present.

Figure S5 compares the time-resolved spectra at two times for samples with initial temperatures of 100 and 175 °C to the corresponding FT-IR difference spectra from the same starting temperature. ΔT values for the FT-IR were chosen to match the temperature increase of the time-resolved sample at the relevant time point, determined as described in the main text, and scaled to correct for different pellet thicknesses by comparison of the < 2000 cm^{-1} framework modes of each sample. The close agreement between time-resolved and FT-IR data is readily apparent, as is the absence of free Brønsted-associated modes at 2660 cm^{-1} in the time-resolved data from 100 °C T_{Init}. The broad hydrogen-bonded background is weaker in the time-resolved data at 10^{-6} s due to the kinetics of water

removal meaning that this mode does not reach full intensity until approximately 10^{-4} seconds (see Section S8).

S7. Deuterated Brønsted Kinetics Providing Evidence for Two Processes at 175 °C

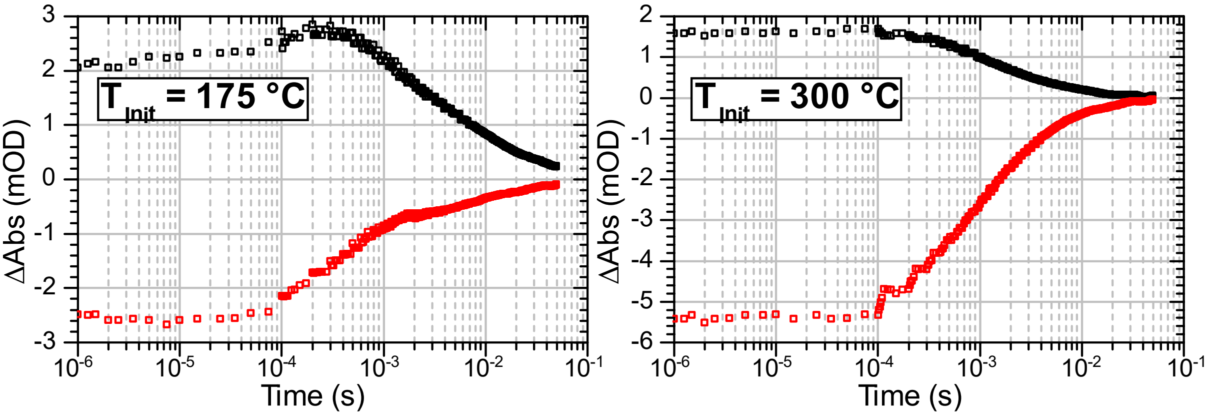

Figure S6: Time-resolved IR spectral intensities at indicated temperatures for positive (black) and negative (red) features in Brønsted ν(ZOD) mode ca. 2660 cm^{-1} after baselining to remove contributions from broad hydrogen-bonded background. Dissimilar kinetics at 175 °C provide evidence for two contributions to the Brønsted ZOD spectrum at this temperature – creation of new free acid sites, and temperature shifting of the frequency of existing free acid sites. At 300 °C, no new Brønsted ZOD groups are formed and the changes observed are due to frequency shifting alone.

S8. Comparison of Silanol and H-Bonded Brønsted Decay Kinetics at Different Temperatures:

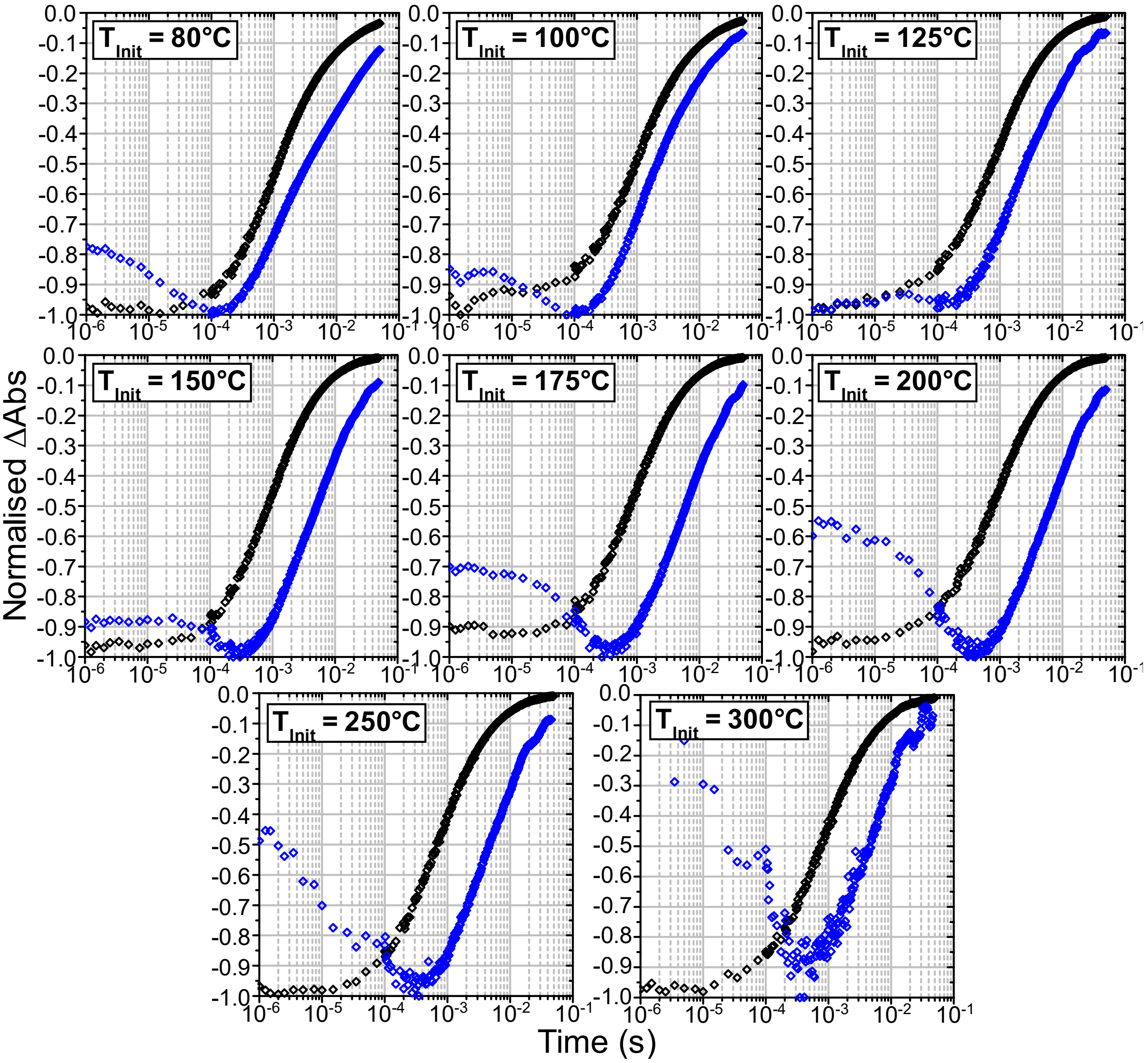

Figure S7: Kinetic plots of normalised change in spectral intensity vs time for silanol ν(SiOD) mode (black) and hydrogen-bonded Brønsted ν(OH) feature at 2925 cm^{-1} (blue).

Figure S7 compares the rates of decay for the zeolite silanol ν(SiOD) mode, representative of the temperature of the zeolite framework, and the negative feature at 2925 cm^{-1}, representing the loss of hydrogen-bonded Brønsted acid sites and thus the level of water desorption. Water readsorption is delayed relative to the zeolite cooling, however the effect is less pronounced at lower temperatures.

S9. Deriving Temperature Change Values from Time-Resolved IR Data

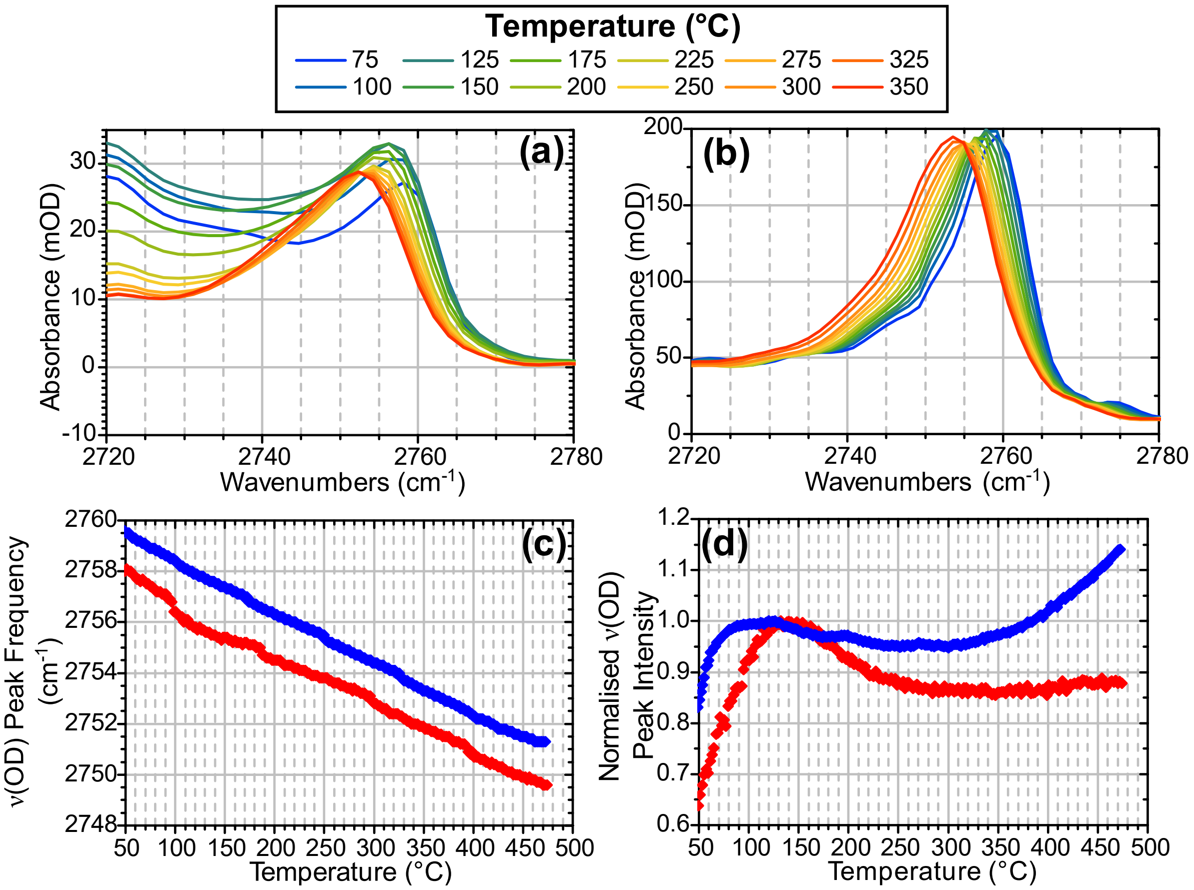

Figure S8: (a,b) FT-IR spectra of ZSM-5 (a) and fumed silica (b) at indicated temperatures. (c) variation of ν(SiOD) peak frequency with temperature for ZSM-5 (red) and silica (blue) from 50 – 475 °C. (d) variation of ν(SiOD) peak intensity with temperature for ZSM-5 (red) and silica (blue) from 50 – 475 °C. Intensities normalised to the value at 120 °C to allow comparison between samples.

When ZSM-5 is heated the silanol stretch modes (located *ca*. 3750 cm^{-1} for SiOH and *ca*. 2755 cm^{-1} for the deuterated SiOD) shift to lower frequency (Figure S8(a)). After increasing in intensity up to *ca.* 120 °C they also weaken until the intensity stabilises at approximately 300 °C. This behaviour is also observed for silanol modes in other materials, such as pyrogenic silica , as illustrated in (Figure S8(b)). The rate of frequency change with temperature is constant and the same for both ZSM-5 and silica, although the silanol modes in silica are positioned approximately 2 cm^{-1} higher in frequency at a given temperature (Figure S8(c)). The weakening of the ν(SiOD) band on heating is more pronounced for ZSM-5 than for pyrogenic silica which also exhibits an increase in ν(SiOD) intensity for temperatures > 300 °C (Figure S8(d)). This is caused by high temperature restructuring and breaking of hydrogen

bonded SiOD groups in the silica, which increases the population of non-hydrogen bonded SiOD groups.

The combination of the shift in frequency and the weakening of the ν(SiOD) mode in ZSM-5 produces the sharp feature observed at 2760 cm^{-1} in the FT-IR difference spectra for heating from a given starting temperature (Figure S3 and Figure 3 in the main text). The weakening of the mode has the effect of suppressing the positive side of the derivative shape usually associated with difference spectra of peak shifts (Figure S9) making the feature mainly negative in intensity in the measured difference spectra.

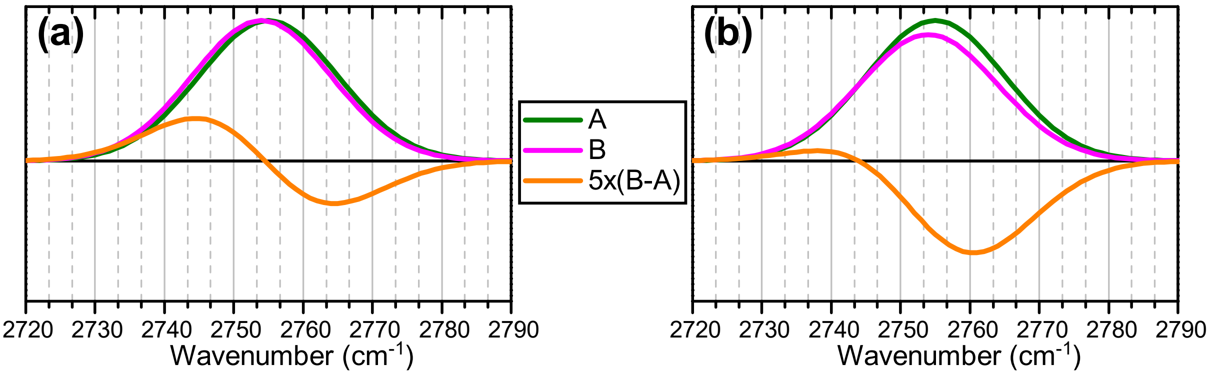

Figure S9: Simulation of the characteristic shape associated with difference spectra involving (a) frequency shifting of a peak and (b) simultaneous shifting and weakening of the peak. The absolute size of the frequency and amplitude shifts match those expected for heating of ZSM-5 from 150 to 250 °C taken from Figure S8(c,d). Amplitude of the difference spectrum is scaled up by 5× for clarity.

By subtracting a baseline passing through points close to the edges of the silanol mode, contributions to the FT-IR difference spectrum from the water desorption are eliminated and the effect of the silanol mode shift isolated, as shown in Figure 5 in the paper. *K* values determined from the calibration plots for each value of T_{Init} were determined through processing their respective difference spectra series and are given in Table S2. The effect of differing pellet thickness between the FT-IR calibration temperature series and T-jump time-resolved IR data collections was corrected for by examining the absorption intensity of the zeolite lattice modes at <2000 cm^{-1} measured by FT-IR for the two different experiments.

Table S2: Fitted values of K for each T_{Init}.

T_{Init} (°C)	*K* (°C/mOD)
80	-1.89×10^4
100	-2.48×10^4
125	-2.75×10^4
150	-2.49×10^4
175	-2.27×10^4
200	-2.23×10^4
250	-2.17×10^4
300	-2.11×10^4

To confirm that the presence of flowing water vapour around the sample does not affect the variation of the silanol mode with temperature, Figure S10 compares the results as described above for T_{Init} = 100 °C (Figure S10, blue) with those for an experiment under identical conditions except that 98% of the flow gas passing through the cell bypasses the HOD/H_2O bubbler, reducing the relative humidity of the cell environment (Figure S10, red). It was not possible to perform this experiment under dry flow gas conditions as trace H_2O contamination of the N_2 supply caused back-exchange of SiOD to SiOH above 150 °C, introducing an additional contribution into the variation of ν(SiOD) with temperature. This back-exchange is still present in the 2% hydrated data presented here, being responsible for the two traces diverging above 200 °C ΔT, but is reduced enough to confirm that the ν(SiOD) behaviour is unaffected by the presence of water across the temperature range of interest.

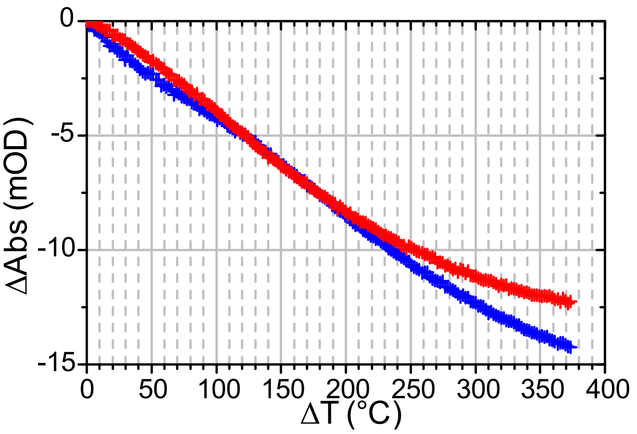

Figure S10: Variation of ν(SiOD) negative intensity with ΔT for ZSM-5 under flowing N_2 saturated with HOD/H_2O (blue) and a flow of HOD/H_2O saturated N_2 diluted 50:1 with additional dry N_2 (red). Total flow rate through the sample cell is the same between samples.

Baseline subtraction of the time-resolved spectra in the same manner as performed for the FT-IR difference spectra allows the variation of the SiOD negative intensity with time to be extracted (Figure S11(a,b)). Multiplying this value at each time point by the appropriate K value converts it to a plot of ΔT *vs*. time (Figure S11(c)), as reported in the main text.

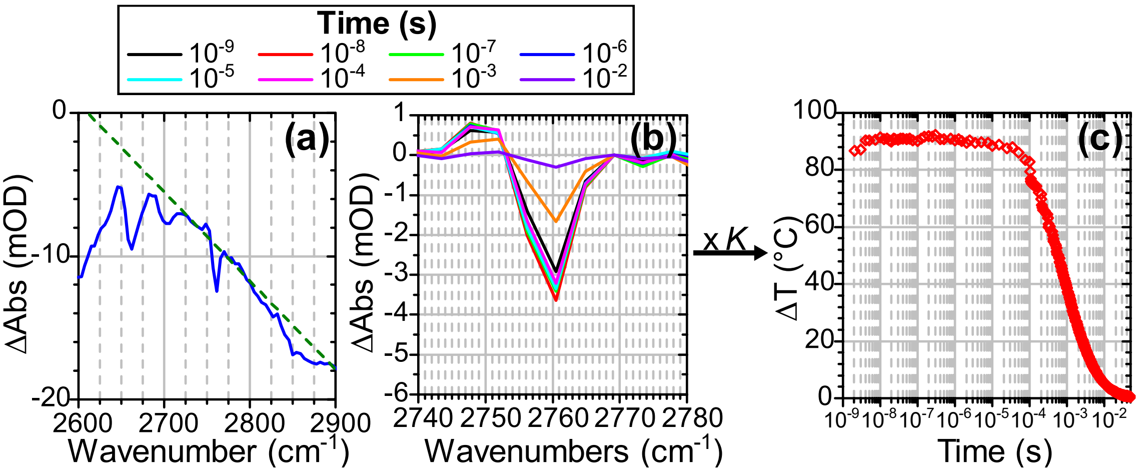

Figure S11: (a) Time resolved spectrum at t = 1 μs following pumping from T_{Init} = 175 °C showing linear baseline fit to isolate silanol mode contributions. (b) Comparison of ν(SiOD) feature in baseline subtracted time resolved spectra at indicated time points showing variation in intensity of negative feature with time. (c) Sample ΔT *vs.* time derived by multiplying the ν(SiOD) negative intensity measured at each time point by conversion factor *K*

S10. Fit Parameters for Cooling Curves

Tables S3 and S4 present the results from fitting the cooling curves of experimental and simulated T-jump data respectively to a bi-exponential decay function.

Table S3: Maximum achieved ΔT and cooling fit parameters for experimental T-jumps from all values of T_{Init}.

T_{Init} (°C)	ΔT_{Max} (°C)	τ_1 (ms)	A_1	τ_2 (ms)	A_2	τ_2/τ_1
**80**	109.0 ± 1.2	1.10 ± 0.02	0.71 ± 0.01	15.4 ± 0.7	0.29 ± 0.01	14.0 ± 0.69
**100**	113.4 ± 3.7	0.98 ± 0.03	0.70 ± 0.01	12.4 ± 0.6	0.3 ± 0.01	12.7 ± 0.73
**125**	144.7 ± 1.8	0.67 ± 0.02	0.65 ± 0.02	6.47 ± 0.3	0.35 ± 0.02	9.66 ± 0.53
**150**	101.7 ± 1.3	0.79 ± 0.02	0.67 ± 0.02	6.72 ± 0.3	0.33 ± 0.01	8.51 ± 0.44
**175**	89.4 ± 3.2	0.83 ± 0.02	0.68 ± 0.02	6.93 ± 0.3	0.32 ± 0.01	8.35 ± 0.41
**200**	68.7 ± 1.3	0.66 ± 0.02	0.63 ± 0.02	5.81 ± 0.3	0.37 ± 0.02	8.80 ± 0.53
**250**	57.9 ± 1.3	0.63 ± 0.02	0.68 ± 0.02	5.93 ± 0.3	0.32 ± 0.01	9.41 ± 0.56
**300**	56.2 ± 0.6	0.69 ± 0.02	0.67 ± 0.02	6.42 ± 0.3	0.33 ± 0.01	9.30 ± 0.51

Table S4: Zeolite thermal conductivity, maximum achieved ΔT and cooling fit parameters for simulated T-jumps from all T_{Init} values. Thermal conductivity determined by comparison of experimental and simulated cooling curves.

T_{Init} (°C)	κ (W m^{-1} K^{-1})	ΔT_{Max} (°C)	τ_1 (ms)	A_1	τ_2 (ms)	A_2	τ_2/τ_1
**80**	-	178.4 ± 60.6	-	-	-	-	-
**100**	-	149.8 ± 53.8	-	-	-	-	-
**125**	0.2 ± 0.1	122.9 ± 48.7	1.32 ± 0.96	0.71 ± 0.32	11.7 ± 8.47	0.29 ± 0.14	8.73 ± 2.3
**150**	0.2 ± 0.1	103.6 ± 44.9	1.31 ± 0.95	0.69 ± 0.34	11.7 ± 8.43	0.31 ± 0.16	8.62 ± 2.36
**175**	0.2 ± 0.1	94.3 ± 39.3	1.25 ± 0.94	0.69 ± 0.26	11.1 ± 7.37	0.31 ± 0.13	8.61 ± 2.02
**200**	0.25 ± 0.1	85.5 ± 38.0	0.97 ± 0.64	0.68 ± 0.34	9.11 ± 6.02	0.32 ± 0.17	8.64 ± 2.50
**250**	0.3 ± 0.1	72.2 ± 38.0	0.82 ± 0.51	0.68 ± 0.39	7.35 ± 4.47	0.32 ± 0.19	8.97 ± 2.45
**300**	0.3 ± 0.1	63.3 ± 34.3	0.91 ± 0.56	0.67 ± 0.38	8.43 ± 5.28	0.33 ± 0.20	8.70 ± 2.36

S11. Equations Used for Zeolite Heat Transfer Simulations

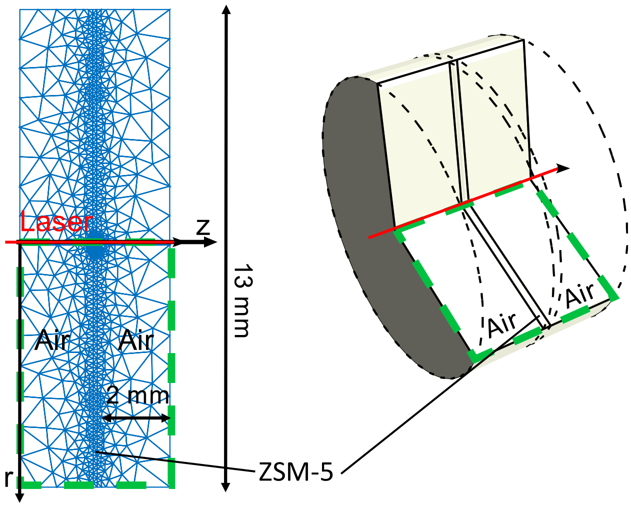

Figure S12: Parameters of a 2D axisymmetric heat equation as applied to zeolite laser heating, showing the coordinate mesh for numerical analysis in solid blue lines and the unique simulated region in dashed green lines (the simulation being axisymmetric).

To understand the time dependent temperature distribution within the ZSM-5 pellets during and following the T-jump laser pulses, the initial temperature profile and its evolution over time were simulated in three dimensions. The model was based on the axisymmetric heat equation in cylindrical coordinates (Equation S1), which was solved numerically using finite element analysis in Mathematica version 13.2.[1]

$$\rho C_p \frac{\partial T}{\partial t} + \frac{1}{r}\frac{\partial}{\partial r}\left(-\kappa r \frac{\partial T}{\partial r}\right) + \frac{\partial}{\partial z}\left(-\kappa \frac{\partial T}{\partial z}\right) = Q \qquad (S1)$$

The ZSM-5 pellet was assumed to be a cylinder sandwiched between cylinders of air, as illustrated in Figure S12. Coordinate terms r and z are those perpendicular and parallel to the laser beam axis respectively. The laser beam profile was modelled as a perfectly circular Gaussian, allowing use of the axisymmetric Equation S1. Parameters ρ, C_p and κ are the density, specific heat capacity and isotropic thermal conductivity and were assigned differently between the ZSM-5 and air regions of the simulation volume. Q is the heat source, i.e. the T-jump laser beam, defined by:

$$Q = (1 - R)10^{-\mu(z - d_{air})} N\mu \ln(10) \qquad (S2)$$

R is the reflection coefficient of T-jump laser light from the front surface of the pellet, μ is the absorbance per unit length of the pump light by the pellet, in units of optical density per meter, d_{air}

is the thickness of the air layer on either side of the pellet (2 mm for simulation purposes) and N is a normalised Gaussian in r, defined by:

$$N = \frac{2E}{\tau_{pulse}\pi w_{pump}(z)^2} e^{-\frac{2r^2}{w_{pump}(z)^2}} \qquad (S3)$$

Where E is the pulse energy of the T-jump laser, τ_{pulse} is the duration of the T-jump pulse and $w_{pump}(z)$ is the z-dependent pump beam waist, defined as the half-width at 1/e^2 intensity.

The heat source was present for the first 1 ns of the simulation only. The laser pulse was assumed to only be absorbed by (and heat) the sample, not the air around it, with the radial distribution being described by the Gaussian spot given by Equation S3. The spot size was assumed to vary over the thickness of the sample according to Gaussian optics. The Gaussian optics equations used calculate the z dependence of the beam waist w, which is related to the full-width at half maximum intensity spot size $FWHM_{pump}$ by:

$$w_{pump} = \frac{FWHM_{pump}}{\sqrt{-2\ln(0.5)}} \qquad (S4)$$

The Rayleigh length z_R is defined in terms of the beam waist as:

$$z_R = \frac{\pi(w_0)^2}{\lambda M^2} \qquad (S5)$$

Where w_0 is the beam waist at the focus, λ is the wavelength and M^2 is the beam quality factor. The size of the spot at a given position is described as a function of the distance from the focus by:

$$w_{pump}(z) = w_0\sqrt{1 + \left(\frac{z}{z_R}\right)^2} \qquad (S6)$$

The diffraction limited spot size of a Gaussian beam is given by:

$$w_0 = \frac{4M^2\lambda f}{\pi D} \qquad (S7)$$

Where f is the focal length of the lens and D is the beam diameter at the lens. Based on the z-dependent beam profile measurements described in ESI Section S3, it was determined that the beam quality factor was likely to lie between 6 and 16, corresponding to a Rayleigh length of ~60-200 µm,

resulting in divergence on the scale of the thickness of the pellet (~100 µm). We comment further on this in Section S16.

The outer boundary of the air layer and the outer edges of the pellet were held at the starting temperature using Dirichlet conditions. The outer layer of air was set to be 2 mm thick, which was found to be sufficient for the results to be unaffected (less than 1% change of the pellet temperature up to 1 second) by further increases in air thickness, or changes in the boundary conditions. The ZSM-5 pellet was set to 13 mm in diameter. This diameter was sufficient that the hot spot did not reach the edge of the modelled region within the simulation time for up to 50 consecutive laser shots at 1 kHz.

The model was solved over the whole of the pellet and the surrounding air. The size of the mesh elements was varied to be finest around the hot region of the pellet, with the area of the triangular elements set to 0.5 µm^2 in this region (Figure S12).

S12. Simulation Input Parameters

Table S5: Average values and bounds for parameters used as inputs in T-jump simulations of water-loaded ZSM-5 pellets.

Pulse energy	71 ± 5 µJ
Pump spot FWHM	36.0 ± 1.4 µm
Probe spot FWHM	21.5 ± 1.0 µm
Absorbance	See Table S6
Pellet thickness	110 ± 5 µm
Density	See Figure S13
Specific heat capacity	See Equation S8
Thermal conductivity	Table S4
Beam quality factor	11 ± 5
Reflection	0.014 ± 0.014

12.1 Zeolite Thermal properties

The density of the ZSM-5 pellets used in the T-Jump experiments was determined by weighing pressed pellets, measuring their thickness using digital vernier callipers and determining their overall area using digitised photographs of the pellets at a known scale, processed in imaging software 'Fiji'.[2] The variation of ZSM-5 mass with temperature due to water desorption was measured using

thermogravimetric analysis (TGA) of the pellets from 20-500 °C. The final density value at each temperature was calculated as an average of 11 measurements, with the results shown in Figure S13.

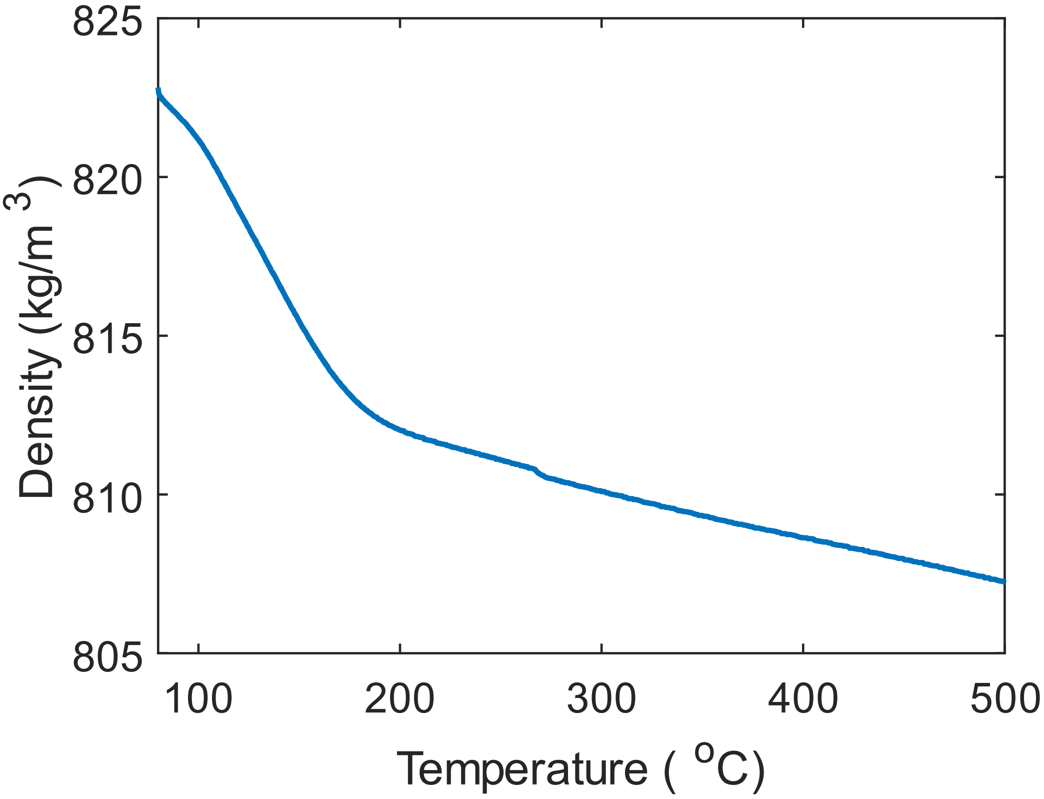

Figure S13: Temperature dependent density of ZSM-5 pellets determined by applying a temperature dependent mass correction to room temperature density measurements

The thermal properties of the air region were defined according to the starting temperature prior to each T-jump, then assumed to stay constant over the course of the heating and cooling process. These values were taken from Mathematica's thermodynamic data lookup tables.[3,4]

The specific heat capacity (C_p) of zeolites is temperature-dependent and also varies both between zeolite framework types and with the Si:Al ratio within a framework type.[5] While the relationship of C_p with framework type and Si:Al is not straightforward, the temperature dependence may be modelled based on fitting of experimental data. For this work we used the second order polynomial relationship determined by Lu et al.[6] for the temperature dependent C_p of H-ZSM-5 with a Si:Al of 23:1 measured from 40 – 180 °C *via* calorimetry. This closely matches our zeolite composition and experimental conditions. This gives Equation S8, which was used to vary C_p with T within each T-jump simulation, resulting in the heat transfer equation Eq S1 becoming nonlinear.

$$C_p(T) = 1194.68 + A(T - 392.77) + B(T^2 - 392.77^2) \qquad (S8)$$

Where A is -3.87 ± 0.75 J kg^{-1} K^{-2} and B is 0.0084 ± 0.001 J kg^{-1} K^{-3}.

Thermal conductivity of MFI-type zeolites is not well defined in the literature, as the majority of studies reported investigate either intracrystalline conductivity[7] or the behaviour in polycrystalline thin films.[8–10] The latter's thermal conductivity may differ significantly from the loosely pressed powder samples considered here due to the influence of differing levels of thermal contact between the individual crystallites. The reported range of values from these literature studies of 0.8 – 1.2 W m^{-1} K^{-1} is therefore best regarded as an upper limit. Thermal conductivity does not have a significant impact on the magnitude of the simulated T-jump, only the cooling time. Derivation of a suitable value for the thermal conductivity at each starting temperature was therefore carried out empirically and is described as part of the discussion of the simulation results in the main text.

12.2 Zeolite Optical Properties

Pellet thickness measurements were made using digital vernier callipers.

The reflection coefficient of ZSM-5 was calculated from the refractive index as reported by Olson et al.[11] using Snell's law.[12]

The T-Jump pump laser spectrum was observed to be around 100 cm^{-1} FWHM, and non-Gaussian in spectral-shape (Figure S14(c)). The amount of pump light absorbed by the sample was accounted for as follows. The absorbance of the pellet was weighted according to the overlap integral between the pump spectrum and the FT-IR spectrum of ZSM-5. To do this, the baselined FT-IR spectrum of ZSM-5 at the starting temperature was converted from optical density units (α) to an attenuation factor A.

$$A = 1 - 10^{-\alpha} \qquad (S9)$$

The overlap integral of the FT-IR spectrum and the pump spectrum was then calculated, with the result taken as the spectrum of the absorbed pump energy. This was normalised against the integral of the original pump spectrum and converted back to optical density units. A long tail in the pump spectrum was the dominating source of uncertainty in these calculations, so calculations including and excluding these portions of the pump spectrum were carried out and the variation used to define the error interval. These calculations were repeated for each starting temperature, with the resulting absorbance values shown in Table S6.

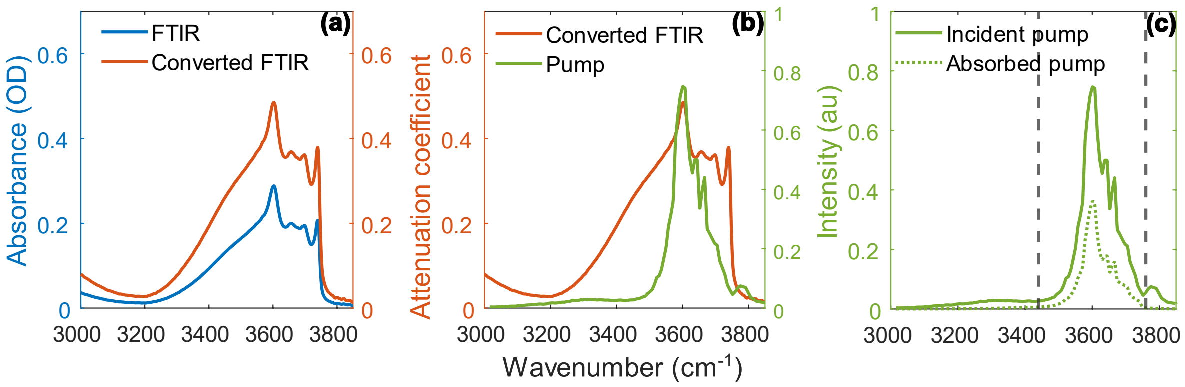

Figure S14: Spectra for ZSM-5 pump absorbance calculations at 175 °C (a) FT-IR spectrum of ZSM-5 at 175 °C and its conversion to attenuation coefficient (b) Overlap of the FT-IR with pump spectrum used to calculate pump absorbance (c) Comparison of spectra of incident pump and absorbed pump energy. The dashed vertical grey lines indicate the regions of the pump spectrum that were excluded for uncertainty calculations

12.3 Pump laser parameters

Pump laser parameters in the simulation were based on those measured experimentally, with a pulse energy of 71 ±5 µJ and beam profile as given in Table S1. As the model used was axisymmetric the average FWHM values were used as the input.

Table S6: Absorption coefficients for each of the ZSM-5 pellet T-Jump starting temperatures, calculated from the overlap integral of FT-IR and pump spectrum.

Temperature (ºC)	Absorbance (OD)
80	0.42 ± 0.03
100	0.32 ± 0.02
125	0.25 ± 0.02
150	0.21 ± 0.02
175	0.20 ± 0.01
200	0.19 ± 0.01
250	0.18 ± 0.02
300	0.18 ±0.01

S13. Experimental Factors Excluded from Simulation Model

The data presented in the main text includes error bars which represent the degree of uncertainty in the input parameters used. However, there were some remaining experimental factors that were not included in the model. Experimental beam profile measurements showed the pump spot to be elliptical but simulations assumed a perfectly circular Gaussian beam to allow use of an axisymmetric model to reduce computational expense. The possibility of variations in the asymmetry of the beam through the thickness of the pellet was also discounted. Inclusion of these parameters would require a more detailed three-dimensional model and a larger series of beam profile measurements.

The effect of scattering was also ignored in simulations. Scattering would, to first order, diffuse the focussed laser spot making it larger than expected as it propagates across the sample. The expected result of including this might be a small reduction (<5%) in average T-jump due to the increase in spot size across the thickness of the pellet, based on the observed effects of including beam divergence in the simulations. The magnitude of the increase in spot size observed experimentally could in future be measured with a thermal camera to allow this to be included in simulations.

The temperature gradient across the pellet due to the heater geometry of the sample cell would not affect the starting temperature of the probed region of the zeolite significantly, due to the hole in the heater block being much larger than the probed region (4 mm : 21.5 μm).[13] The presence of the heater block itself and the possible temperature gradient in the air surrounding the zeolite prior to pumping were not considered. The magnitude of these effects is likely to be negligible due to the low contribution of cooling in the z direction to the overall cooling behaviour, provided the probed region is well centred in the hole.

As mentioned in the main text, the cooling of laser heated zeolites pellets in air is dominated by the conduction of heat into the cooler regions of the zeolite, with conduction into the surrounding air playing a minor role. The effects of cooling through convection or radiation in the simulations were also determined to be of minimal importance (Figure S15). While typical heat transfer coefficients for free convection into air vary from 0.5 – 1000 W m^{-2} K^{-1},[14–16] it has been reported that on the microscale this can be enhanced to as much as 7000 W m^{-2} K^{-1}.[17,18] Simulations using a convective boundary condition and a heat transfer coefficient of 10,000 W m^{-2} K^{-1} were found to give similar results to simulations assuming pure conduction into surrounding air, with small (<10%) deviations in temperature on timescales <1 ms. Implementing a radiative boundary condition that assumes the zeolite pellet is a perfect blackbody also had no effect on the overall cooling rate.

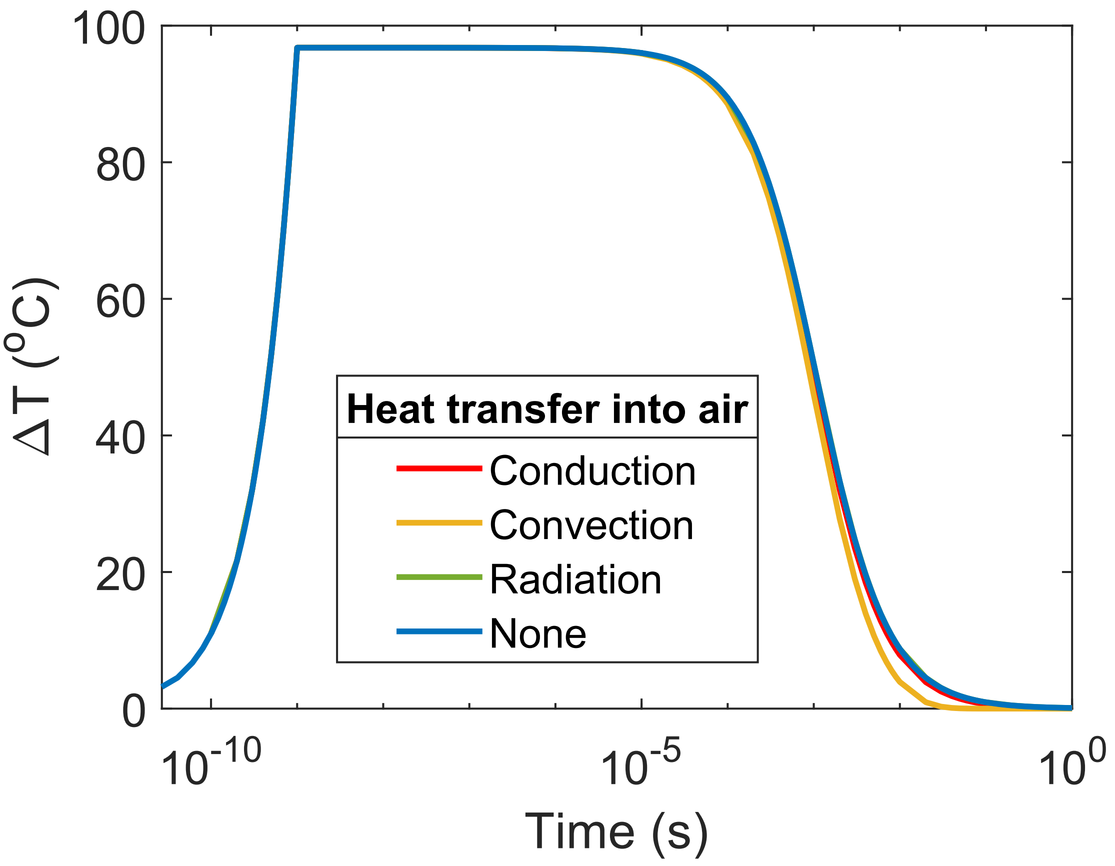

Figure S15: Simulated cooling curves for a laser heated ZSM-5 pellet demonstrating the effect of different types of heat transfer into the surrounding air. Heat transfer by conduction into the unheated zeolite material was allowed in all cases.

In all simulations, only C_p is fully temperature dependent. Although the absorbance of the sample varied with starting temperature, it was assumed to stay constant throughout the heating and cooling process of each T-jump. The same was true of the density of the zeolite. Thermal conductivity was a free parameter and defined separately for each starting temperature. However, this value was not allowed to vary during the heating and cooling process.

Finally, the simulations assume that the pump and probe beams are perfectly aligned, with their maximum intensities being in the same position. Any deviation from this would result in experimentally observed T-jumps which are lower than expected.

S14. Obtaining temperature-time curves from simulations by spatial averaging

The axisymmetric temperature distribution was first multiplied by a Gaussian of amplitude 1, with its width defined by the probe spot size to account for the weighting due to sampling by the probe. The result was then integrated over the cylindrical region that represented the part of the sample within the FWHM of the probe beam, and the result of this integral divided by the volume of this region, as well as a normalisation factor related to the initial Gaussian weighting.

$$\frac{\int_{d_{air}}^{d_{air}+d}\int_0^{FWHM_{probe}} T(t,r,z)e^{-\frac{2r^2}{w_{probe}^2}}rdrdz}{\int_{d_{air}}^{d_{air}+d}\int_0^{FWHM_{probe}} e^{-\frac{2r^2}{w_{probe}^2}}rdrdz} \quad (S10)$$

S15. Comparing Simulations of Water Heating with Literature Data

To test the validity of the model used, the results of simulations of water sandwiched between CaF_2 windows were compared to results reported in literature. Water simulations carried out by Chung et al.[19] use a similar treatment as this work. The radial temperature distribution was assumed to be Gaussian, and heat transfer was calculated in both r and z directions. The initial temperature change was assumed to be constant with z due to the low sample absorbance. Simulations carried out by Greetham et al.[20] assumed heat transfer in the z direction only. This is a reasonable approximation, as the thermal conductivity of CaF_2 is much greater than that of water. The probe beam in this case was significantly smaller than the pump beam and radial cooling was assumed to be insignificant.

To allow comparison with the results of Greetham et al.,[20] the same simulation parameters were used – the pump spot size was taken as 300 µm (FWHM), the pump pulse energy as 70 µJ, the path length of the water as 10 µm and the absorbance of water as 0.5 OD. The windows were modelled as 5 µm thick, with Dirichlet boundary conditions on the outer surface, setting the temperature to 0 ^{o}C. The thermal properties of water and CaF_2 were matched to those used in the literature. Reflection and divergence of the beam were not included, and all thermal properties were assumed to be entirely independent of temperature. The result of the Mathematica simulations based on Equation S1 and simulations of Greetham are presented in Figure S16. Overall, agreement is good. The two models show similar temperature profiles in z, but with the model of the present work giving a lower overall temperature jump magnitude. This is due to the Greetham simulations using a top hat radial distribution of the pump beam and the simulations of the present work being Gaussian, with the pulse

energy spread out over a larger sample area. Simulations based on Equation S1 but using a top hat distribution give a close match to the data of Greetham (Figure S16(a)). The cooling process is shown in Figure S16(b). Simulations using Equation S1 cool slightly slower than the simulations of Greetham, especially at the water-window boundaries, with a greater temperature increase occurring within the windows.

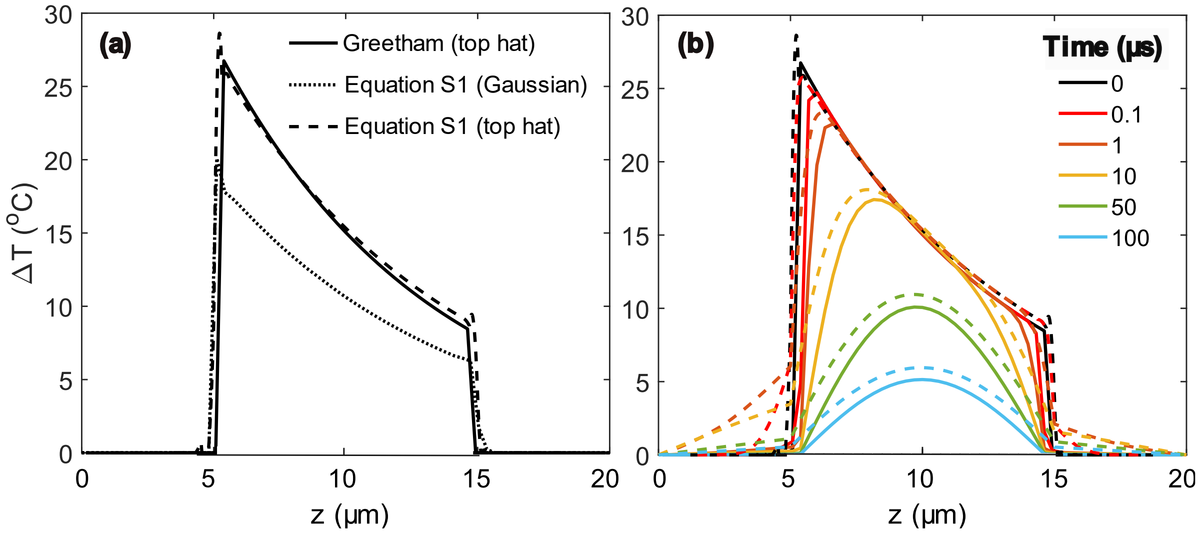

Figure S16: Simulated z dependent temperature distributions of laser heated water sandwiched between CaF_2 windows. Solid lines represent 1D simulated data from literature,[20] while dashed and dotted lines show simulations performed using Eq S1 for different pump beam profiles (a) immediately after a 1 ns laser pulse (b) at various time delays during the cooling process

To allow comparison with the results of Chung et al.,[19] in our simulations, the pulse energy was taken as 5 mJ, the spot size as 500 μm (full width at $1/e^2$ intensity), the absorbance of the water as 0.022 OD, the pellet thickness as 50 μm and the CaF_2 thickness as 1 mm. The T-jump magnitude was in good agreement with both the previously simulated and experimental data. The spatial temperature distributions obtained from these simulations are shown in Figure S17.

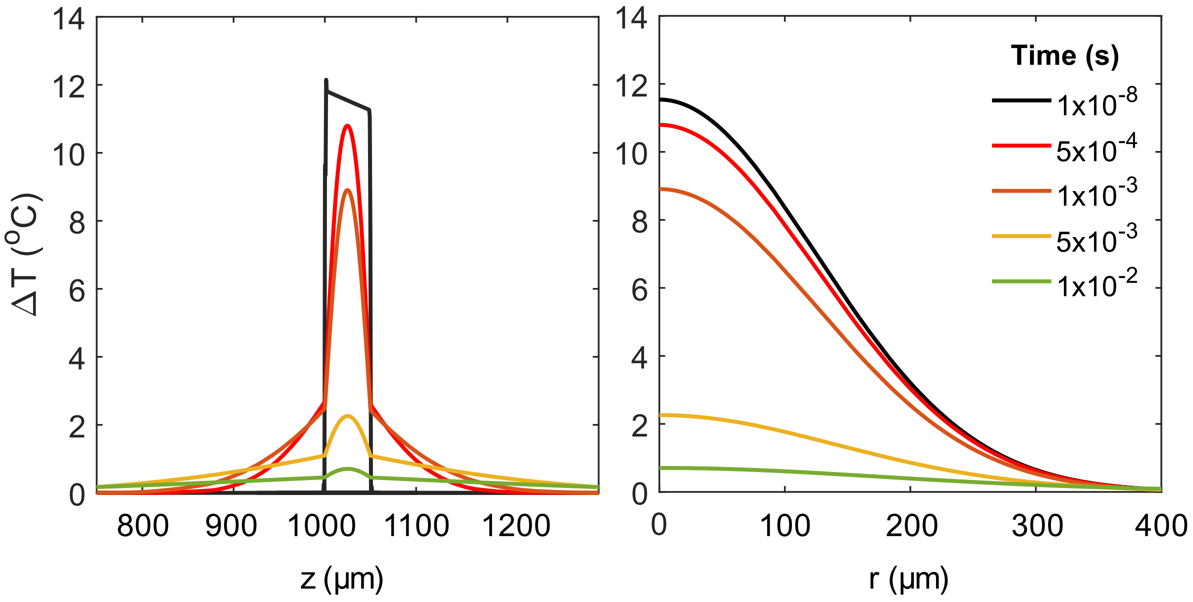

Figure S17: Simulated temperature distributions and their temporal evolution for water sandwiched between CaF_2 windows, with parameters matched to experimental conditions reported by Chung et al[19]

Cooling curves were obtained by averaging the temperature over z at r=0, the same method used in the literature.[19,20] Results from simulating both top hat and Gaussian beam profiles are shown, compared to previously reported experimental and simulated results from Greetham[20], in Figure S18. The experimental cooling timescales were slower than those simulated with 5 μm windows. This is likely due to the CaF_2 windows not acting as perfect heat sinks in experiments. Simulations based on Equation S1 with thicker, 200 μm windows showed an increase in the cooling timescale, also presented in Figure S18.

The cooling curve obtained from simulations showed some deviation from the experimental data presented by Chung[19] on the 100 μs to 1 ms timescale. Differences were also observed compared to the literature simulation results, on similar timescales (Figure S19). This was determined not to be related to the difference in z-dependence of the initial temperature distribution.

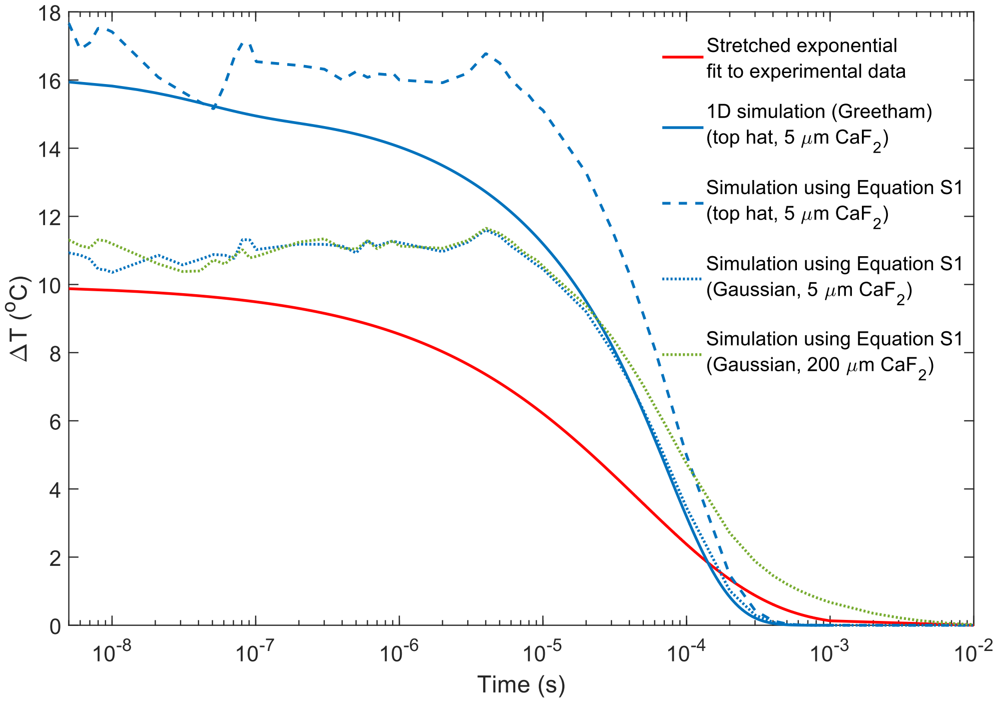

Figure S18: Cooling curves showing experimental and simulated literature data[20] (solid lines) compared to simulations using Equation S1 (dashed and dotted lines) for water sandwiched between CaF_2 windows.

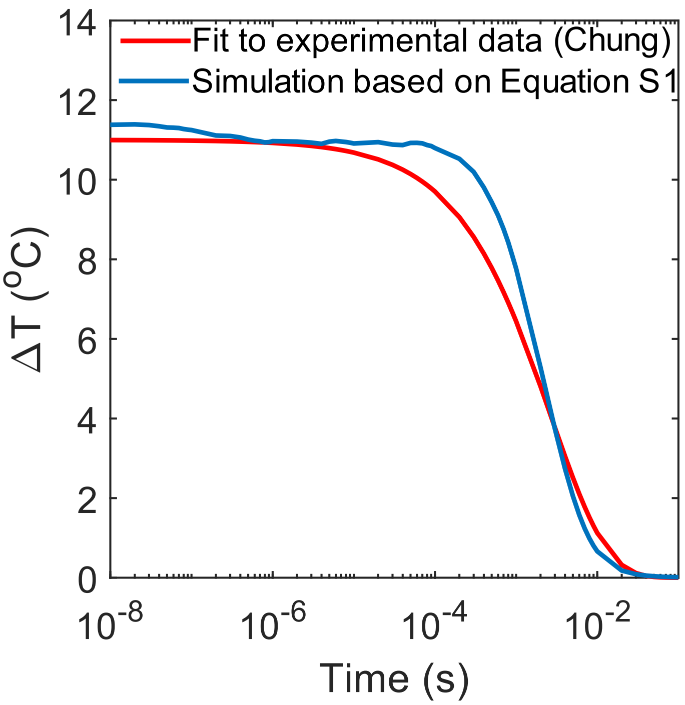

Figure S19: Cooling curves comparing experimental literature data[19] to data simulated using Equation S1, both for water sandwiched between CaF_2 windows.

S16. Comparing Simulations of Zeolite Heating with Literature Data

Sample heating effects have been observed and quantified in picosecond pulsed-IR experiments on Mordenite by Bonn et al.[21] By observing a temperature dependent shift in probe transmission, an increase in temperature of 10-30°C from a single pump pulse was determined. Using the model of the present work, with parameters adjusted to match those used by Bonn et al., a temperature increase of ~18°C is predicted, in good agreement with experimental values. The simulated pulse was 20 ps in duration with 150 µJ pulse energy and 0.4 mm beam waist diameter ($1/e^2$). The absorbance of the sample was assumed to be 0.22 OD, corresponding to pumping at the absorption maximum. The thickness of the pellet was calculated as 24 µm using the area density (5 mg/cm^3)[21] and the volume density of Mordenite (2100 kg/m^3).[22] The beam quality factor of the pump was assumed to be 1, with all remaining parameters assumed to match those used for ZSM-5 simulations.

S17. Divergence of Pump Beam

The divergence of the pump beam was determined by beam profile measurements taken at known distances from the focus, using Gaussian optics equations, as described in Section S3 and S10. The Rayleigh length was found to be somewhere between 60-200 µm. The resulting divergence, which was used to calculate the initial temperature distribution in simulations, is shown in Figure S20.

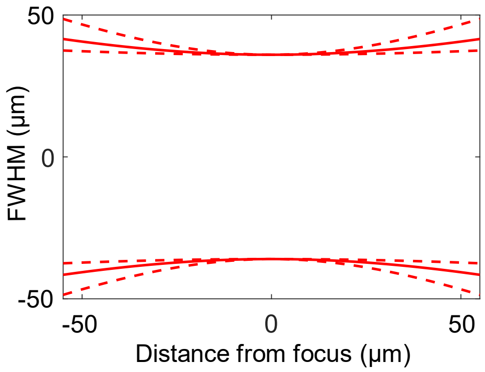

Figure S20: FWHM pump beam width as a function of distance from the focus, showing the divergence calculated from z dependent beam profile measurements and Gaussian optics equations. The solid line shows the divergence based on the mean beam quality factor, with the dashed lines showing the upper and lower bounds.

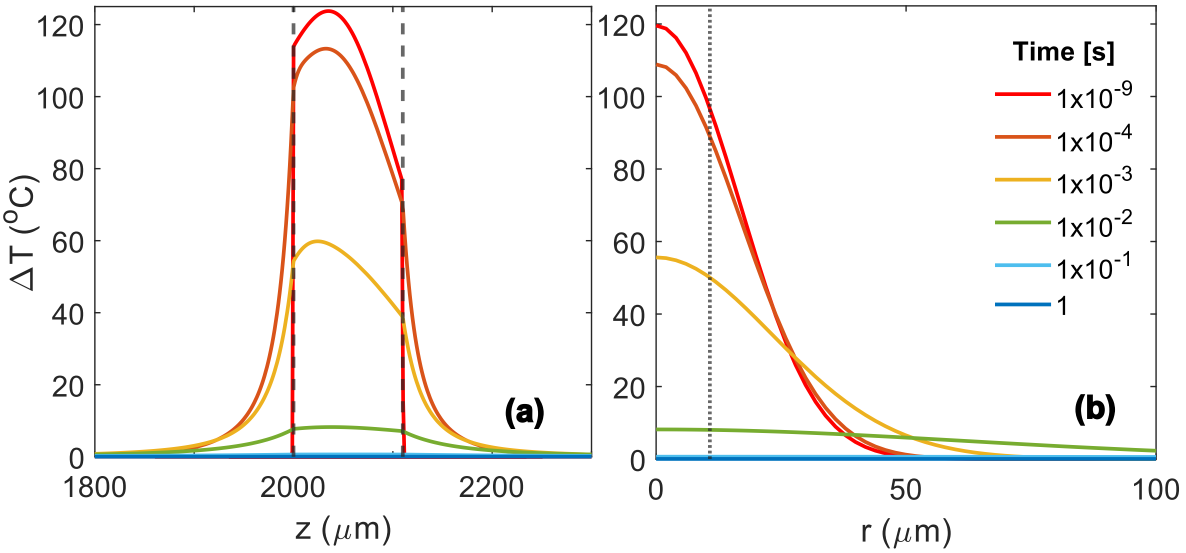

Figure S21: Simulated temperature distributions and their temporal evolution for a T-jump from 175 °C. (a) z-dependent temperature distribution at r = 0. Dashed lines show the boundaries of the ZSM-5 pellet. (b) Radial distribution of the T-jump at the centre of the pellet (z = 2055 μm): dotted line shows the position of the half maximum intensity of a perfectly aligned probe spot.

Figure S21 shows simulated temperature distributions resulting from heating a ZSM-5 pellet with a divergent Gaussian pump beam. As noted in the main text, divergence of the pump beam lowers the intensity at the faces of the pellet, resulting in the highest temperatures in the sample being located in the first half of the pellet depth. Simulations of the cooling process show this inhomogeneity decays with time, but persists to > 1 ms after initial heating.

S18. Deriving Heat Capacity from Experimental Data

The maximum theoretical temperature jump achievable under given conditions is given by the amount of energy E absorbed into a sample of density (ρ), volume (V), OD and heat capacity (C_p) by:

$$\Delta T = \frac{E_{pump} \times OD}{\rho \times V \times \mathrm{C_p}} \qquad (S11)$$

Since the OD values at each temperature are known (Table S6) it is possible to calculate values for the heat capacities based on the experimental ΔT values. The heated volume of the sample in this case was calculated using the Gaussian divergence profile of the pump beam described by Equation S6, but with the beam FWHM as the diameter of the heated zone to account for the fact that the experiment's probe beam mainly observes the centre of the pump beam profile. The length of the heated volume was taken as the pellet thickness (Table S5) and the final volume given by:

$$V = \int_{-5.5\times10^{-5}}^{5.5\times10^{-5}} \pi \left[\frac{FWHM\sqrt{1+\left({}^{z}/{}_{z_R}\right)^2}}{2}\right]^2 dz \qquad (S12)$$

The results of this calculation are discussed in the main text. We note that the major source of uncertainty in the final values comes from beam quality factor of the pump (Table S5).

S19. Investigating the Origins of Biexponential Cooling

As noted in the main text, the cooling of the simulated sample duplicates the bi-exponential cooling behaviour observed experimentally, and this is therefore a heat transfer effect. The ability to examine the contribution of individual heat transfer processes in the simulation to the overall cooling behaviour allows the source of the bi-exponential cooling to be identified as arising from the inhomogeneous thermal energy distribution within the pumped region of the zeolite.

Simulation of zeolite cooling under vacuum conditions by setting the thermal conductivity of the air region of the simulation volume to zero did not noticeably affect the cooling behaviour, indicating that heat transfer from the zeolite into the surrounding air is not significant and the heated zone cools radially by conduction into the rest of the zeolite pellet.

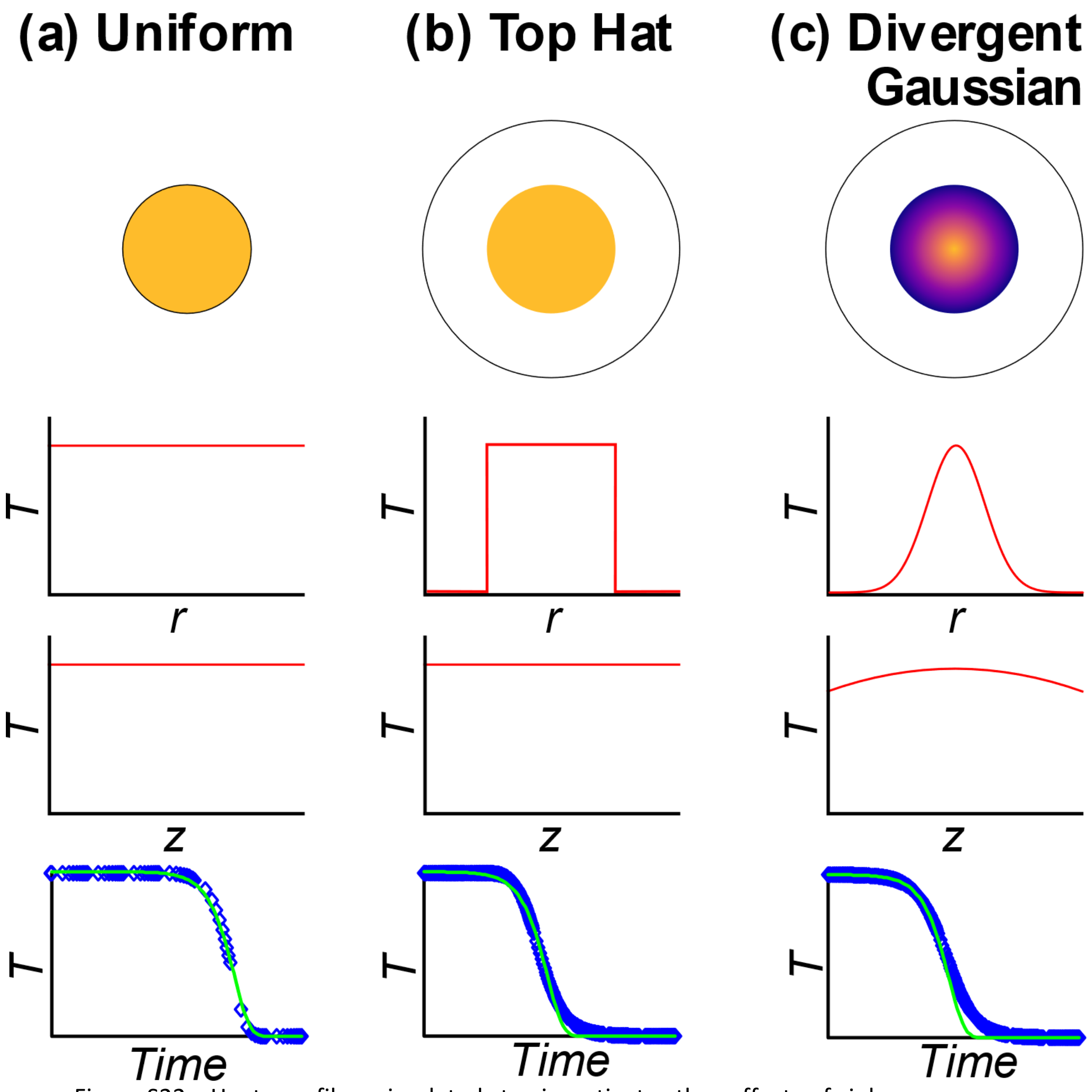

Figure S22: Heat profiles simulated to investigate the effect of inhomogeneous temperature distribution from laser pumping on sample cooling behaviour. Circles represent the zeolite volume in the simulation, which are surrounded by air in all cases. White regions of the zeolite are not heated by the laser pulse but can heat through conduction as the sample evolves. Radial and z-axis temperature profiles represent the sample state immediately after completion of the laser heating. Temperature vs time curves (blue) show cooling of each simulation. The green line represents a single-exponential fit of the cooling, showing close agreement in the case of simulation (a) and deviations from this behaviour for simulations (b) and (c).

Figure S22 shows the results of simulating different types of heat distribution to investigate their effect on cooling. Simulating a uniformly-heated zeolite body surrounded by air in all dimensions ('Uniform', Figure S22(a)) results in a single-exponential cooling curve. The cooling of this sample was slower than that observed in the other simulations due to the lower heat transfer into air compared with

conduction into zeolite (Table S7). Simulation of a uniformly-heated zeolite zone surrounded by cool ZSM-5 material in the *r* dimension ('Top Hat', Figure S22(b)) results in the reintroduction of bi-exponential cooling as a temperature gradient forms within the heated region due to the exterior cooling faster than the interior. Introduction of non-uniform temperature distributions in the heated zone due to Gaussian beam shape and divergence ('Divergent Gaussian' Figure S22(c)) have the effect of increasing the relative contribution of the 'slow' exponential term to the overall cooling behaviour, indicating that the magnitude of A_2 is determined by the degree of inhomogeneity in the heat distribution (Table S7).

Exponential cooling is an outcome of Newton's law of cooling, valid under a number of simplifying assumptions.[23] It assumes that both the thermal conductivity and heat capacity of the cooling body are independent of temperature, which is not the case in the system under investigation. It also requires that the cooling body is thermally 'thin', or in other words, that cooling occurs equally at all points within the hot zone. This latter condition requires that the thermal conductivity of the hot zone be high in comparison to the thermal conductivity of the surroundings into which it cools. Due to the dominance of radial conductive cooling for the case of laser heated zeolites, the hot body in this case is primarily cooling into material with identical thermal conductivity and so the thin body requirement is violated. Under these kinds of conditions, it is known that the actual cooling behaviour will exhibit distortions away from Newtonian single-exponential cooling behaviour towards various kinds of distorted exponential curves, including bi-exponential behaviour.[24–26]

Table S7: Fit parameters for cooling of simulations with temperature profiles as shown in Figure S22.

**Simulation Heat Profile**	**τ_1 (ms)**	***A_1***	**τ_2 (ms)**	***A_2***	**τ_2/τ_1**
**Uniform**	13.1 ± 0.25	1.00	-	0.00	-
**Top Hat**	0.23 ± 0.01	0.85 ± 0.01	2.68 ± 0.2	0.15 ± 0.01	11.65 ± 1.00
**Divergent Gaussian**	0.83 ± 0.02	0.68 ± 0.02	6.93 ± 0.3	0.32 ± 0.01	8.35 ± 0.41

S20. Computer Simulations of Multi-Pump Heating

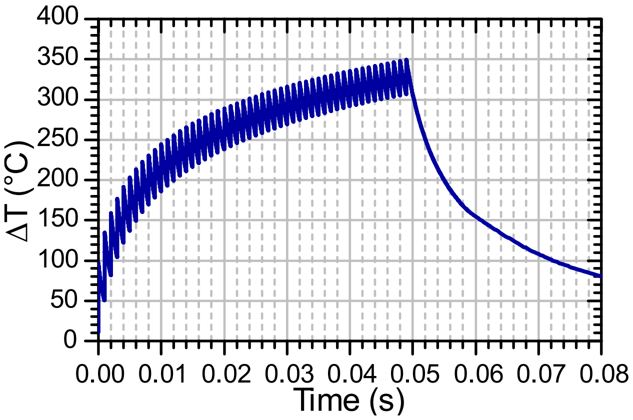

Figure S23: Average sample temperature vs time for simulation of a HOD in ZSM-5 sample at an initial temperature of 175 °C subjected to 50 × 70 µJ pump pulses at 1 kHz repetition rate.

Figure S23 shows the simulated temperature profile over time for a ZSM-5 sample excited by a burst of 50 pump pulses at 1 kHz from an initial temperature of 175 °C. Compared to the experimental case discussed in the main text, this simulation assumes a pump OPO wavelength of 2.8 µm, leading to higher absorbance of the pump light. This results in a larger increase in temperature per pulse and demonstrates that the sample can achieve higher ΔT values with multi-pump heating than were observed experimentally for single-pulse heating at 2.8 µm (main text, Figure 6).

Figure S24 shows the spatial temperature distribution at various pulse positions in the same 50 pulse burst shown in Figure S23. In the z direction, the inhomogeneity due to pump absorbance and divergence becomes more extreme with subsequent pulses. In the radial direction, the heated region gets larger with an increased number of pulses, effectively reducing the thermal gradient in this direction. As radial conduction is dominant in the cooling process, this has a significant effect on cooling time.

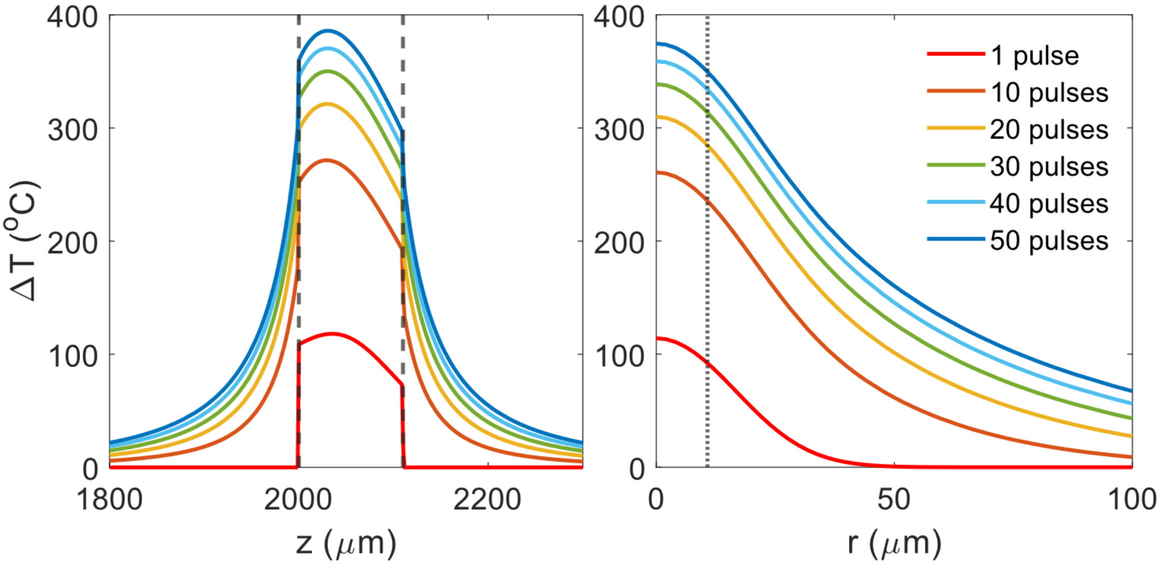

Figure S24: Simulated temperature distributions for a T-jump from 175 °C following indicated pulse number in a 50 pulse burst (a) z-dependent temperature distribution at r = 0. Dashed lines show the boundaries of the ZSM-5 pellet. (b) Radial distribution of the T-jump at the centre of the pellet (z = 2055 μm): dotted line shows the position of the half maximum intensity of a perfectly aligned probe spot.

References

(1) Wolfram Research, Inc. Mathematica, 2022.

(2) Schindelin, J.; Arganda-Carreras, I.; Frise, E.; Kaynig, V.; Longair, M.; Pietzsch, T.; Preibisch, S.; Rueden, C.; Saalfeld, S.; Schmid, B.; Tinevez, J.-Y.; White, D. J.; Hartenstein, V.; Eliceiri, K.; Tomancak, P.; Cardona, A. Fiji: An Open-Source Platform for Biological-Image Analysis. *Nat Methods* **2012**, *9* (7), 676–682. https://doi.org/10.1038/nmeth.2019.

(3) Wolfram|Alpha Curated Data, 2022.

(4) Lemmon, E.W., Huber, M.L., McLinden, M.O. NIST Standard Reference Database 23: Reference Fluid Thermodynamic and Transport Properties-REFPROP, Version 9.1, National Institute of Standards and Technology, Standard Reference Data Program, Gaithersburg, 2013.

(5) Voskov, A. L.; Voronin, G. F.; Kutsenok, I. B.; Kozin, N. Yu. Thermodynamic Database of Zeolites and New Method of Their Thermodynamic Properties Evaluation for a Wide Temperature Range. *Calphad* **2019**, *66*, 101623. https://doi.org/10.1016/j.calphad.2019.04.008.

(6) Lu, X.; Wang, Y.; Estel, L.; Kumar, N.; Grénman, H.; Leveneur, S. Evolution of Specific Heat Capacity with Temperature for Typical Supports Used for Heterogeneous Catalysts. *Processes* **2020**, *8* (8), 911. https://doi.org/10.3390/pr8080911.

(7) Hudiono, Y.; Greenstein, A.; Saha-Kuete, C.; Olson, B.; Graham, S.; Nair, S. Effects of Composition and Phonon Scattering Mechanisms on Thermal Transport in MFI Zeolite Films. *J. Appl. Phys. (Melville, NY, U. S.)* **2007**, *102* (5), 053523. https://doi.org/10.1063/1.2776006.

(8) Greenstein, A. M.; Graham, S.; Hudiono, Y. C.; Nair, S. Thermal Properties and Lattice Dynamics of Polycrystalline MFI Zeolite Films. *Nanoscale Microscale Thermophys. Eng.* **2006**, *10* (4), 321–331. https://doi.org/10.1080/15567260601009171.

(9) Coquil, T.; Lew, C. M.; Yan, Y.; Pilon, L. Thermal Conductivity of Pure Silica MEL and MFI Zeolite Thin Films. *J. Appl. Phys. (Melville, NY, U. S.)* **2010**, *108* (4), 044902. https://doi.org/10.1063/1.3462500.

(10) Fang, J.; Huang, Y.; Lew, C. M.; Yan, Y.; Pilon, L. Temperature Dependent Thermal Conductivity of Pure Silica MEL and MFI Zeolite Thin Films. *J. Appl. Phys. (Melville, NY, U. S.)* **2012**, *111* (5), 054910. https://doi.org/10.1063/1.3692754.

(11) Olson, D. H.; Haag, W. O.; Lago, R. M. Chemical and Physical Properties of the ZSM-5 Substitutional Series. *Journal of Catalysis* **1979**, No. 61, 390–396.

(12) Woan, Graham. *The Cambridge Handbook of Physics Formulas*; Cambridge University Press: Cambridge, UK, 2000.

(13) Donaldson, P. M.; Howe, R. F.; Hawkins, A. P.; Towrie, M.; Greetham, G. M. Ultrafast 2D-IR Spectroscopy of Intensely Optically Scattering Pelleted Solid Catalysts. *The Journal of Chemical Physics* **2023**, *158* (11), 114201. https://doi.org/10.1063/5.0139103.

(14) *Convective Heat Transfer*. https://www.engineeringtoolbox.com/convective-heat-transfer-d_430.html (accessed 2023-08-22).

(15) *Convective Heat Transfer Coefficients Table Chart*. https://www.engineersedge.com/heat_transfer/convective_heat_transfer_coefficients__13378.htm (accessed 2023-08-22).

(16) *Convection Heat Coefficient*. https://help.solidworks.com/2015/english/SolidWorks/cworks/c_convection_heat_coefficient.htm?format=P&value= (accessed 2023-08-22).

(17) Lin, L.; Wu, H.; Xue, L.; Shen, H.; Huang, H.; Chen, L. Heat Transfer Scale Effect Analysis and Parameter Measurement of an Electrothermal Microgripper. *Micromachines* **2021**, *12* (3), 309. https://doi.org/10.3390/mi12030309.

(18) Pulavarthy, R. S. R. A. Characterization of Heat Transfer Coefficient at Micro/Nano Scale and the Effect of Heated Zone Size, The Pennsylvania State University, 2015.

(19) Chung, H. S.; Khalil, M.; Smith, A. W.; Tokmakoff, A. Transient Two-Dimensional IR Spectrometer for Probing Nanosecond Temperature-Jump Kinetics. *Rev. Sci. Instrum.* **2007**, *78* (6), 063101. https://doi.org/10.1063/1.2743168.

(20) Greetham, G. M.; Clark, I. P.; Young, B.; Fritsch, R.; Minnes, L.; Hunt, N. T.; Towrie, M. Time-Resolved Temperature-Jump Infrared Spectroscopy at a High Repetition Rate. *Appl. Spectrosc.* **2020**, *74* (6), 720–727. https://doi.org/10.1177/0003702820913636.

(21) Bonn, M.; Brugmans, M. J. P.; Kleyn, A. W.; Van Santen, R. A. Fast Energy Delocalization upon Vibrational Relaxation of a Deuterated Zeolite Surface Hydroxyl. *The Journal of Chemical Physics* **1995**, *102* (5), 2181–2186. https://doi.org/10.1063/1.468740.

(22) Oprea, C.; Popescu, V.; Birghila, S. New Studies about the Modified Mordenites. *Rom. Journ. Phys.* **2008**, *53*, 231–239.

(23) da Silva, S. L. E. F. Newton's Cooling Law in Generalised Statistical Mechanics. *Physica A: Statistical Mechanics and its Applications* **2021**, *565*, 125539. https://doi.org/10.1016/j.physa.2020.125539.

(24) Mondol, A.; Gupta, R.; Das, S.; Dutta, T. An Insight into Newton's Cooling Law Using Fractional Calculus. *J. Appl. Phys. (Melville, NY, U. S.)* **2018**, *123* (6), 064901. https://doi.org/10.1063/1.4998236.

(25) Brown, A.; Marshall, T. K. Body Temperature as a Means of Estimating the Time of Death. *Forensic Sci.* **1974**, *4*, 125–133. https://doi.org/10.1016/0300-9432(74)90093-4.

(26) Davidzon, M. I. Newton's Law of Cooling and Its Interpretation. *Int. J. Heat Mass Transfer* **2012**, *55* (21), 5397–5402. https://doi.org/10.1016/j.ijheatmasstransfer.2012.03.035.

(27) Schnell, S. K.; Vlugt, T. J. H. Thermal Conductivity in Zeolites Studied by Non-Equilibrium Molecular Dynamics Simulations. *Int. J. Thermophys.* **2013**, *34* (7), 1197–1213. https://doi.org/10.1007/s10765-013-1467-2.
